# Regularity of Solutions to the Muskat Equation

**DOI:** 10.1007/s00205-023-01862-z

**Published:** 2023-04-10

**Authors:** Jia Shi

**Affiliations:** Department of Mathematics, Massachusetts Institute of Technology, Simons Building (Building 2), Room 157, Cambridge, MA 02139 USA

## Abstract

In this paper, we show that if a solution to the Muskat problem in the case of different densities and the same viscosity is sufficiently smooth, then it must be analytic except at the points where a turnover of the fluids happens.

## Introduction

The Muskat problem is a free boundary problem studying the interface between fluids in the porous media [30]. It can also describe the Hele–Shaw cell [34]. The density function $\rho$ follows the active scalar equation

1.1 $$\begin{aligned} \frac{\text {d}\rho }{dt}+(v\cdot \nabla )\rho =0, \end{aligned}$$

with

$$\begin{aligned} \rho (x,t)=\left\{ \begin{array}{ccc} \rho _1 &{}&{} x\in D_1(t),\\ \rho _2 &{}&{} x\in D_2(t). \end{array}\right. \end{aligned}$$

Here $D_1(t)$ and $D_2(t)$ are open domains with $D_1(t)\cup D_2(t) \cup \partial D_{1}(t)=\mathbb {R}^2$. The velocity field *v* in (1.1) satisfies Darcy’s law,

1.2 $$\begin{aligned} \frac{\mu }{\kappa }v=-\nabla p -(0,g\rho ), \end{aligned}$$

and the incompressibility condition

$$\begin{aligned} \nabla \cdot v=0, \end{aligned}$$

where *p* is the pressure and $\mu$ is the viscosity. $\kappa$, *g* are the permeability constant and the gravity force.

We focus on the problem where two fluids have different densities $\rho _1, \rho _2$ and the same viscosity $\mu$.

After scaling, the equation for the boundary $\partial D_{1}(t)$ in the periodic setting read as

1.3 $$\begin{aligned} \frac{\partial f_i}{\partial t}(\alpha ,t)=\frac{\rho _2-\rho _1}{2}\int _{-\pi }^{\pi } \frac{\sin (f_1(\alpha )-f_1(\alpha -\beta ))(\partial _{\alpha }f_i(\alpha )-\partial _{\alpha }f_i(\alpha -\beta ))}{\cosh (f_2(\alpha )-f_2(\alpha -\beta ))-\cos (f_{1}(\alpha )-f_{1}(\alpha -\beta ))}\text {d}\beta \end{aligned}$$

for $i=1,2$ (see [10]). Here $f(\alpha ,t)=(f_1(\alpha ,t),f_2(\alpha ,t))$ is a parameterization of the boundary curve. $f(\alpha ,t)-(\alpha ,0)$ is periodic in $\alpha$.

**Fig. 1:**
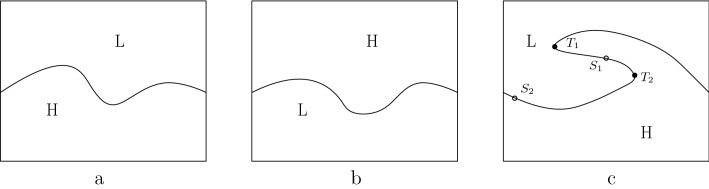
Three regimes of the Muskat equation

Given an initial interface at time 0, (1.3) is divided into three regimes. When the interface is a graph and the heavier fluid is on the bottom as in Fig. 1a, it is in a stable regime. When heavier fluid is above the boundary as in Fig. 1b, it is in a stable regime when time flows backward. Thus, given any initial data, (1.3) can be solved for small negative time *t*. In both regimes, shown in Fig. 1a, b, (1.3) can not be solved in the wrong direction unless the initial interface is real analytic. The third regime, shown in Fig. 1c, it highly unstable because the heavier fluid lies on top near point $S_1$ while the lighter fluid lies on top near point $S_2$. Note two turnover points $T_1$ and $T_2$ where the interface has a vertical tangent. For generic initial data in the turnover regime, (1.3) has no solutions either as time flows forward or backward.

In the third regime, there are several examples from the literature (eg. [9, 10, 19, 20]), but they are all real analytic solutions. Without the real analytic assumption, due to the spatially non-consistent parabolic behavior, the existence is usually false and the uniqueness is unknown. To address this gap, this paper studies to what extent the solution of (1.3) is analytic.

Moreover, for the analytic solutions, one can prove an energy estimate on an analyticity region that shrinks when time increases. That energy estimate implies uniqueness in the class of analytic solutions. [10]. Therefore, the investigation towards analyticity can serve as a first step to deal with the uniqueness.

We introduce a new way to prove that any sufficiently smooth solution is analytic except at the turnover points. Here is our main theorem:

### Theorem 1.1

Let $f(\alpha ,t)=(f_1(\alpha ,t),f_2(\alpha ,t))\in C^{1}([-t_0,t_0],H^{6}[-\pi ,\pi ]\times H^{6}[-\pi ,\pi ]))$ be a solution of the Muskat equation (1.3) satisfying the arc-chord condition. If $\partial _{\alpha }f_1(\alpha _0,t)\ne 0$, and $-t_0< t<t_0$, then $f(\cdot ,t)$ is analytic at $\alpha _0$.

Our method concerning the analyticity is not limited to the Muskat problem. A simplified version of our method can be used to show the analyticity of the solution to a kind of non-local differential equations (see Section 10). This approach is new to our best knowledge.

In our forthcoming work [35], we focus on the degenerate analyticity near the turnover points. The existence and uniqueness are crucially related to the way the real-analyticity degenerates at those points. Given an extra assumption, we have the following theorem in [35]:

### Theorem 1.2

Let $f(\alpha ,t)=(f_1(\alpha ,t),f_2(\alpha ,t))\in C^{1}([-t_0,t_0],C^{100}([-\pi .\pi ])$ be a solution of the Muskat equation (1.3) with two turnover points. $Z_1(t)$, $Z_2(t)$ are values of $\alpha$ of these two turnover points. If we assume that the solution satisfies the following three conditions:

1.4 $$\begin{aligned}&\partial _{\alpha }^2f_1(Z_1(t),t)\ne 0, \end{aligned}$$

1.5 $$\begin{aligned}&\partial _{\alpha }f_1(\alpha ,t) \ne 0 \text { except at}\, Z_1(t), Z_2(t), \end{aligned}$$

and

1.6 $$\begin{aligned}&\left( \frac{d Z_1}{dt}(t)+\frac{\rho _2-\rho _1}{2}p.v.\int _{-\pi }^{\pi }\frac{\sin (f_1(\alpha ) -f_1(\alpha -\beta ))}{\cosh (f_2(\alpha )-f_2(\alpha -\beta ))-\cos (f_1(\alpha )-f_1(\alpha -\beta ))}\text {d}\beta \right) \nonumber \\&\quad \frac{\rho _2-\rho _1}{2}\partial _{\alpha }^2f_1(Z_1(t),t)<0, \end{aligned}$$

then when $-t_0<t<t_0$, $f(\cdot ,t)$ can be analytically extended to region $\Omega =\{x+iy|-\epsilon _1(t)+Z_{1}(t)\le x\le Z_{1}(t)+\epsilon _1(t),|y|\le \epsilon _2(t)(x-Z_{1}(t))^2\}$.

### Background

In order to make the equation well-defined, the arc-chord condition is introduced, saying that

$$\begin{aligned} F(f)=\left| \frac{\beta ^2}{\cosh (f_2(\alpha )-f_2(\alpha -\beta ))-\cos (f_{1}(\alpha )-f_{1}(\alpha -\beta ))}\right| \end{aligned}$$

is in $$L^{\infty }$$.

The Rayleigh-Taylor coefficient $\sigma$ is used to characterize the three regimes in Fig. 1 and is defined as

1.7 $$\begin{aligned} \sigma =\frac{\rho _2-\rho _1}{2}\frac{\partial _{\alpha }f_{1}(\alpha ,t)}{(\partial _{\alpha }f_1(\alpha ,t))^2+(\partial _{\alpha }f_2(\alpha ,t))^2}. \end{aligned}$$

$\sigma \ge 0$ is corresponding to the stable regime and $\sigma \le 0$ the backward stable regime. When $\sigma$ changes sign, it is in the unstable regime.

In the stable regime (heavier liquid is below the lighter liquid), local well-posedness and the global well-posedness with constraints on the initial data have been widely studied, with the lowest space $H^{\frac{3}{2}}$ ( [1–7, 13–17, 21–25, 29, 31, 32, 36, 38, 39]). The existence of self-similar solutions has also been proved [28]. Interesting readers can see [13, 28] for detailed reviews. Due to the parabolic behavior, instant analyticity has been proved in the stable regime. Castro–Córdoba–Fefferman–Gancedo–López-Fernández [10] proved the $H^4$ solutions become instantly analytic if the solutions remain to be in the stable region for a short time. In [29], also in the stable region, Matioc improved the instant analyticity to $H^{s}$, where $s\in (\frac{3}{2},3)$. In [27], Gancedo–García-Juárez–Patel–Strain showed that in the stable regime, a medium size initial data in $\dot{\mathcal {F}^{1,1}}\cap L^2$ with $\Vert f\Vert _{\mathcal {F}^{1,1}}=\int |\zeta ||\hat{f}(\zeta )|\text {d}\zeta$ becomes instantly analytic. Their result also covers the different viscosities case and the 3D case.

When the heavier liquid is above the lighter liquid, the equation is ill-posed when time flows forward [22].

A solution that starts from a stable regime and develops turnover points was first discovered in [10]. That solution still exists for a short time after turnover due to the analyticity when the turnover happens. Moreover, breakdown of smoothness can happen [9]. There are also examples where the solutions transform from stable to unstable and go back to stable [19] and vice versa [20].

Weak solutions and a special kind of weak solutions: mixing solutions of (1.1) have also been studied. They do not satisfy (1.3) and can develop a mixing zone. Weak solutions do not have uniqueness [18]. In all three regimes, there are infinitely many mixing solutions ( [8, 11, 12, 26, 33, 37]).

### The outline of the Proof of Theorem 1.1

Inspired by the instant analyticity results in the stable case [10, 27, 29], our first idea is localization. If locally the lighter liquid is over the heavier one, we let the time go forward, and if locally the heavier one is over the lighter one, we let the time go backward.

Since it leads to lots of difficulties by the standard method due to the localization, we use a new idea to prove analyticity except at turnover points. The idea is to make a $C^1$ continuation of the parametrized interface $\alpha \rightarrow (f_1(\alpha ,t),f_2(\alpha ,t))$ to complex $\alpha$ and then prove the $C^1$ continuation satisfies the Cauchy-Riemann equation. To do so, we break the complex region into curves $\alpha +ic(\alpha )\gamma t$ with $\gamma \in [-1,1]$. On each such curve, we solve an equation for $(f_1,f_2).$ We then show that when $\gamma$ varies, our solutions on the curve fit together into an $C^1$ function of $\alpha +i\beta$. Finally, we prove that $C^1$ function satisfies the Cauchy-Riemann equation, thus producing the desired analytic continuation.

In Section 3, we define a cut off function $\lambda (\alpha )$ and focus on $f^c(\alpha ,t)=\lambda (\alpha )f(\alpha ,t)$. We then localize the equation such that the modified *R*-*T* condition has a fixed sign. In order to make use of the sign, if the sign is positive, we let the time go forward. If the sign is negative, we let the time go backward.

**Fig. 2:**
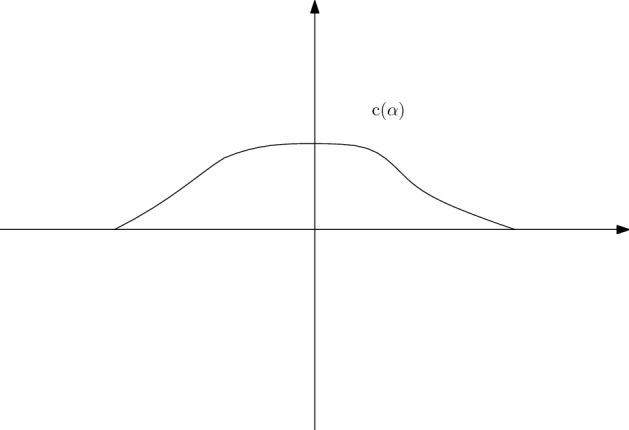
The curve $c(\alpha )$ for Theorem 1.1

In Section 4, we introduce $c(\alpha )$ with $\textrm{supp} c(\alpha )\subset \{\alpha |\lambda (\alpha )=1\}$ (Fig. 2). With the assumption that $f^c(\alpha ,t)$ is analytic in domain $D_A=\{\alpha +i\beta |-c(\alpha )t\le \beta \le c(\alpha )t\}$, we derive the equation on the curve $\{(\alpha +ic(\alpha )\gamma t)|\alpha \in [-\pi ,\pi ]\}$ for fixed $\gamma \in [-1,1]$. Then we obtain the equation

1.8 $$\begin{aligned} \frac{d}{dt}z(\alpha ,\gamma ,t)=T(z(\alpha ,\gamma ,t),t), \end{aligned}$$

with $z(\alpha ,\gamma ,0)=f^c(\alpha ,0)$. The analyticity assumption on $f^c$ is dropped after we get (1.8).

In Section 5, for each fixed $\gamma$, we use the energy estimate and the Galerkin method to show the existence of the solution $z(\alpha ,\gamma ,t)$. The main term is controlled by Gårding’s inequality, where we use a lemma from [9]. This part is similar as to [9, 10].

In Sections 6, 7, and 8, we verify that the $z(\alpha ,0,t)$ coincides with the $f^c(\alpha ,t)$ and that $z(\alpha ,\gamma ,t)$ is also smooth enough with respect to $\gamma$.

In Section 9, we derive some lemmas about the Cauchy-Riemann operator and use those lemmas to show analyticity of $z(\alpha ,\frac{\beta }{c(\alpha )t}\gamma ,t)$ by checking that it satisfies the Cauchy-Riemann equations.

#### Remark 1.3

In [10], the analyticity domain can be chosen as a strip, and the analyticity follows directly from existence. Since our $c(\alpha )$ is supported in a small region, we do not have such good behavior.

## Notation

In the paper we will use the following notations:

$\delta$: a sufficiently small number.

$\lambda (\alpha )$: $\lambda (\alpha )\ge 0$ and in $C^{100}(-\infty ,\infty )$, satisfying

$$\begin{aligned} \lambda (\alpha )={\left\{ \begin{array}{ll}1&{}|\alpha |\le \delta ,\\ 0&{}|\alpha |\ge 2\delta . \end{array}\right. } \end{aligned}$$

$\delta _c$: sufficiently small number depending on $\delta$.

$$c(\alpha )$$:

$$\begin{aligned} \Bigg \{\begin{array}{cc} c(\alpha )=\delta _c , \text { when }|\alpha | \le \frac{\delta }{32},\\ \textrm{supp} c(\alpha ) \subset [-\frac{\delta }{8},\frac{\delta }{8}],\\ c(\alpha )\ge 0, c(\alpha ) \in C^{100}(-\infty ,\infty ), \Vert c(\alpha )\Vert _{C^{100}(-\infty ,\infty )}\le \delta . \end{array} \end{aligned}$$

$f(\alpha ,t)=(f_1(\alpha ,t),f_2(\alpha ,t))$: the original solution of the Muskat equation.

$$f^c(\alpha ,t)$$, $$\tilde{f}(\alpha ,t)$$:

$$\begin{aligned} f^c(\alpha ,t)= & {} \lambda (\alpha )f(\alpha ,t),\\ \tilde{f}(\alpha ,t)= & {} (1-\lambda (\alpha ))f(\alpha ,t). \end{aligned}$$

$t_0$: the original solution exists when $t\in [-t_0,t_0]$.

$$D_A$$: $$D_A=\{(\alpha +i\beta )|-\infty< \alpha < \infty , -c(\alpha )t\le \beta \le c(\alpha )t\}.$$

For any vector function $z =(z_1,z_2)\in H^{k}$: $z_1\in H^{k}$ and $z_2\in H^{k}$.

## The Localization

This step is to localize the equation such that the *R*-*T* coefficient has a fixed sign. Without loss of generality, we study the behavior at origin and let $\frac{\rho _2-\rho _1}{2}=1$. Let $\lambda (\alpha ) \in C^{100}(-\infty ,\infty )$ satisfying $\lambda (\alpha )\ge 0$ and

$$\begin{aligned} \lambda (\alpha )={\left\{ \begin{array}{ll}1&{}|\alpha |\le \delta ,\\ 0&{}|\alpha |\ge 2\delta , \end{array}\right. } \end{aligned}$$

and $f^{c}(\alpha ,t)=f(\alpha ,t)\lambda (\alpha )$, $\tilde{f}(\alpha ,t)=f(\alpha ,t)(1-\lambda (\alpha ))$. Here $\delta$ is a sufficiently small number such that when $\alpha \in [-2\delta ,2\delta ]$, $\partial _{\alpha }f_1(\alpha ,0)$ has a fixed sign. Without loss of generality, we assume

3.1 $$\begin{aligned} \partial _{\alpha }f_1(\alpha ,0)>0. \end{aligned}$$

Then we have

3.2 $$\begin{aligned} \frac{\partial f^c_{\mu }}{\partial t}&=\lambda (\alpha )\int _{-\pi }^{\pi } \frac{\sin (f_1^{c}(\alpha )-f_1^c(\beta )+\tilde{f_1}(\alpha )-\tilde{f_1}(\beta ))(\partial _{\alpha }f^c_{\mu }(\alpha )-\partial _{\alpha }f^c_{\mu }(\beta ))}{\cosh (f_2^c(\alpha )-f_2^c(\beta )+\tilde{f}_2(\alpha )-\tilde{f_2}(\beta ))-\cos (f_{1}^c(\alpha )-f_{1}^c(\beta )+\tilde{f_1}(\alpha )-\tilde{f_1}(\beta )))}\text {d}\beta \nonumber \\&\quad +\lambda (\alpha )\int _{-\pi }^{\pi } \frac{\sin (f_1^{c}(\alpha )-f_1^c(\beta )+\tilde{f_1}(\alpha )-\tilde{f_1}(\beta ))(\partial _{\alpha }\tilde{f}_{\mu }(\alpha )-\partial _{\alpha }\tilde{f}_{\mu }(\beta ))}{\cosh (f_2^c(\alpha )-f_2^c(\beta )+\tilde{f}_2(\alpha )-\tilde{f_2}(\beta ))-\cos (f_{1}^c(\alpha )-f_{1}^c(\beta )+\tilde{f_1}(\alpha )-\tilde{f_1}(\beta )))}\text {d}\beta . \end{aligned}$$

We have $f^c\in C^1([0,t_0], (H^6(\mathbb {T}))^2)$, $\tilde{f}-(\alpha ,0)\in C^1([0,t_0], (H^6(\mathbb {T}))^2)$. Here $\mathbb {T}$ is the torus of 2$\pi$.

## The Equation on the Complex Plane

### Change the contour

Let $$c(\alpha )$$ satisfy

4.1 $$\begin{aligned} \Bigg \{\begin{array}{cc} c(\alpha )=\delta _c , \text { when }|\alpha | \le \frac{\delta }{32},\\ \textrm{supp} c(\alpha ) \subset [-\frac{\delta }{8},\frac{\delta }{8}],\\ c(\alpha )\ge 0, c(\alpha ) \in C^{100}(-\infty ,\infty ), \Vert c(\alpha )\Vert _{C^{100}(-\infty ,\infty )}\le \delta . \end{array} \end{aligned}$$

Here $c(\alpha )$ is defined such that $\tilde{f}, \lambda$ can be analytically extended to the complex domain $D_A=\{(\alpha +i\beta )|-\infty< \alpha < \infty , -c(\alpha )t\le \beta \le c(\alpha )t\}$ and satisfy

4.2 $$\begin{aligned} \tilde{f}(\alpha +ic(\alpha )\gamma t,t)=\tilde{f}(\alpha ,t), \end{aligned}$$

and

4.3 $$\begin{aligned} \lambda (\alpha +ic(\alpha )\gamma t)=\lambda (\alpha ), \end{aligned}$$

for any $$\gamma \in [-1,1].$$

Now we assume $f^c$ is also analytic in this complex domain $D_A$. For any fixed $\gamma \in [-1,1]$, we want to find the new equation on the contour $\{\alpha +ic(\alpha )\gamma t|\alpha \in [-\pi ,\pi ]\}$. Let $\alpha _{\gamma }^t=\alpha +ic(\alpha )\gamma t$. We have

4.4 $$\begin{aligned}&\frac{d f_{\mu }^c(\alpha _{\gamma }^t, t)}{dt}=ic(\alpha )\gamma (\partial _{\alpha }f_{\mu }^c)(\alpha _{\gamma }^t, t)+(\partial _{t}f_{\mu }^c)(\alpha _{\gamma }^t, t)\nonumber \\&=ic(\alpha )\gamma (\partial _{\alpha }f_{\mu }^c)(\alpha _{\gamma }^t, t)\nonumber \\&\quad +\lambda (\alpha _{\gamma }^t)\int _{-\pi }^{\pi } \frac{\sin (f_1^{c}(\alpha _{\gamma }^t,t)-f_1^c(\beta _{\gamma }^t,t)+\tilde{f_1}(\alpha _{\gamma }^t,t)-\tilde{f_1}(\beta _{\gamma }^t,t))((\partial _{\alpha }f^{c}_{\mu })(\alpha _{\gamma }^t,t)-(\partial _{\beta }f^{c}_{\mu })(\beta _{\gamma }^t,t))}{\cosh (f_2^{c}(\alpha _{\gamma }^t,t)-f_2^c(\beta _{\gamma }^t,t)+\tilde{f_2}(\alpha _{\gamma }^t,t)-\tilde{f_2}(\beta _{\gamma }^t,t))-\cos (f_1^{c}(\alpha _{\gamma }^t,t)-f_1^c(\beta _{\gamma }^t,t)+\tilde{f_1}(\alpha _{\gamma }^t,t)-\tilde{f_1}(\beta _{\gamma }^t,t))}\nonumber \\&\quad (1+ic'(\beta )\gamma t)\text {d}\beta \nonumber \\&\quad +\lambda (\alpha _{\gamma }^t)\int _{-\pi }^{\pi } \frac{\sin (f_1^{c}(\alpha _{\gamma }^t,t)-f_1^c(\beta _{\gamma }^t,t)+\tilde{f_1}(\alpha _{\gamma }^t,t)-\tilde{f_1}(\beta _{\gamma }^t,t))((\partial _{\alpha }\tilde{f_{\mu }})(\alpha _{\gamma }^t,t)-(\partial _{\beta }\tilde{f_{\mu }})(\beta _{\gamma }^t,t))}{\cosh (f_2^{c}(\alpha _{\gamma }^t,t)-f_2^c(\beta _{\gamma }^t,t)+\tilde{f_2}(\alpha _{\gamma }^t,t)-\tilde{f_2}(\beta _{\gamma }^t,t))-\cos (f_1^{c}(\alpha _{\gamma }^t,t)-f_1^c(\beta _{\gamma }^t,t)+\tilde{f_1}(\alpha _{\gamma }^t,t)-\tilde{f_1}(\beta _{\gamma }^t,t))}\nonumber \\&\quad (1+ic'(\beta )\gamma t)\text {d}\beta . \end{aligned}$$

### The equation on the curve

Let $z(\alpha ,\gamma ,t)$ be the solution of the equation (4.4) with initial data $z(\alpha ,\gamma ,0)=f^c(\alpha ,0)$. Our motivation is to set $z(\alpha ,\gamma ,t)=f^{c}(\alpha +ic(\alpha )\gamma t,t).$ Since

$$\begin{aligned} (\partial _{\alpha }f^c_{\mu })(\alpha _{\gamma }^t,t)=\frac{\partial _{\alpha }(f^c_{\mu }(\alpha _{\gamma }^t,t))}{1+ic'(\alpha )\gamma t}, \end{aligned}$$

we have

4.5 $$\begin{aligned}&\frac{d z_{\mu }(\alpha , \gamma ,t)}{dt}=\frac{ic(\alpha )\gamma }{1+ic^{'}(\alpha )\gamma t}\partial _{\alpha }z_{\mu }(\alpha ,\gamma ,t)\nonumber \\&\quad +\lambda (\alpha _{\gamma }^{t})\int _{-\pi }^{\pi } \frac{\sin (z_{1}(\alpha ,\gamma ,t)-z_{1}(\beta ,\gamma ,t)+\tilde{f_1}(\alpha _{\gamma }^t,t)-\tilde{f_1}(\beta _{\gamma }^t,t))(\frac{\partial _{\alpha }z_{\mu }(\alpha ,\gamma ,t)}{1+ic'(\alpha )\gamma t}-\frac{\partial _{\beta }z_{\mu }(\beta ,\gamma ,t)}{1+ic'(\beta )\gamma t})(1+ic'(\beta )\gamma t)\text {d}\beta }{\cosh (z_{2}(\alpha ,\gamma ,t)-z_{2}(\beta ,\gamma ,t)+\tilde{f_2}(\alpha _{\gamma }^t,t)-\tilde{f_2}(\beta _{\gamma }^t,t))-\cos (z_{1}(\alpha ,\gamma ,t)-z_{1}(\beta ,\gamma ,t)+\tilde{f_1}(\alpha _{\gamma }^t,t)-\tilde{f_1}(\beta _{\gamma }^t,t))}\nonumber \\&\quad +\lambda (\alpha _{\gamma }^t)\int _{-\pi }^{\pi } \frac{\sin (z_{1}(\alpha ,\gamma ,t)-z_{1}(\beta ,\gamma ,t)+\tilde{f_1}(\alpha _{\gamma }^t,t)-\tilde{f_1}(\beta _{\gamma }^t,t))((\partial _{\alpha }\tilde{f_{\mu }})(\alpha _{\gamma }^t,t)-(\partial _{\beta }\tilde{f_{\mu }})(\beta _{\gamma }^t,t))(1+ic'(\beta )\gamma t)\text {d}\beta }{\cosh (z_{2}(\alpha ,\gamma ,t)-z_{2}(\beta ,\gamma ,t)+\tilde{f_2}(\alpha _{\gamma }^t,t)-\tilde{f_2}(\beta _{\gamma }^t,t))-\cos (z_{1}(\alpha ,\gamma ,t)-z_{1}(\beta ,\gamma ,t)+\tilde{f_1}(\alpha _{\gamma }^t,t)-\tilde{f_1}(\beta _{\gamma }^t,t))} \end{aligned}$$

with

$$\begin{aligned} z(\alpha ,\gamma ,0)=f(\alpha ,0). \end{aligned}$$

We drop the analyticity assumption of $f^c$ from now. Notice that $\tilde{f}$ and $\lambda$ can still be analytically extended to $D_A$ as in (4.2) and (4.3).

## The Existence of z for Fixed $\gamma$

### Energy estimate

We first assume *z* is of finite Fourier modes here and do the energy estimate. The idea of the energy estimate is similar as in [9, 10].

Since $\tilde{f}(\alpha +ic(\alpha )\gamma t,t)=\tilde{f}(\alpha ,t)$, $\lambda (\alpha +ic(\alpha )\gamma t)=\lambda (\alpha )$, we have

5.1 $$\begin{aligned} \frac{d z_{\mu }(\alpha ,\gamma ,t)}{dt}&=T(z)=\frac{ic(\alpha )\gamma }{1+ic^{'}(\alpha )\gamma t}\partial _{\alpha }z_{\mu }(\alpha )\nonumber \\&\quad +\lambda (\alpha )\int _{-\pi }^{\pi } \frac{\sin (z_{1}(\alpha )-z_{1}(\beta )+\tilde{f_1}(\alpha ) -\tilde{f_1}(\beta ))\left( \frac{\partial _{\alpha }z_{\mu }(\alpha )}{1+ic'(\alpha )\gamma t}-\frac{\partial _{\beta }z_{\mu }(\beta )}{1+ic'(\beta )\gamma t}\right) (1+ic'(\beta )\gamma t)}{\cosh (z_{2}(\alpha )-z_{2}(\beta )+\tilde{f_2}(\alpha )-\tilde{f_2}(\beta ))-\cos (z_{1}(\alpha )-z_{1}(\beta )+\tilde{f_1}(\alpha )-\tilde{f_1}(\beta ))}\text {d}\beta \nonumber \\&\quad +\lambda (\alpha )\int _{-\pi }^{\pi } \frac{\sin (z_{1}(\alpha )-z_{1}(\beta )+\tilde{f_1}(\alpha )-\tilde{f_1}(\beta ))((\partial _{\alpha }\tilde{f_{\mu }})(\alpha )-(\partial _{\beta }\tilde{f_{\mu }})(\beta ))(1+ic'(\beta )\gamma t)}{\cosh (z_{2}(\alpha )-z_{2}(\beta )+\tilde{f_2}(\alpha )-\tilde{f_2}(\beta ))-\cos (z_{1}(\alpha )-z_{1}(\beta )+\tilde{f_1}(\alpha )-\tilde{f_1}(\beta ))}\text {d}\beta . \end{aligned}$$

Here we omit the dependency of *z* on $\gamma$ and *t*, and the dependency of $\tilde{f}$ on *t* for the sake of simplicity. Let

$$\begin{aligned} U=(H^5(\mathbb {T}))^2, \end{aligned}$$

where $\mathbb {T}$ is the torus of length $2\pi$ and

$$\begin{aligned} \Vert z\Vert _{Arc}=\sup _{\alpha \in [-2\delta ,2\delta ],\beta \in [-\pi ,\pi ]}\frac{(\alpha -\beta )^2}{|\cosh (z_{2}(\alpha )-z_{2}(\beta )+\tilde{f_1}(\alpha )-\tilde{f_1}(\beta ))-\cos (z_{1}(\alpha )-z_{1}(\beta )+\tilde{f_1}(\alpha )-\tilde{f_1}(\beta ))|}. \end{aligned}$$

For the $L^2$ norm, we have

$$\begin{aligned} \Vert T(z)\Vert _{L^2[-\pi ,\pi ]}\lesssim \Vert T(z)\Vert _{L^\infty [-\pi ,\pi ]}\lesssim C(\Vert z\Vert _{U})\Vert z\Vert _{Arc}. \end{aligned}$$

Here *C* is a bounded function depending on $\delta$, $\delta _c$ and $\Vert f\Vert _{C^1([0,t],(H^6[-\pi ,\pi ])^2)}$. We will keep using the same notation *C* in the following proof.

Now we take 5th derivative and have

$$\begin{aligned}&\partial _{\alpha }^5T(z)=\frac{ic(\alpha )\gamma }{1+ic^{'}(\alpha )\gamma t}\partial _{\alpha }^{6}z_{\mu }(\alpha )\\&\quad +\lambda (\alpha )\int _{-\pi }^{\pi } \frac{\sin (z_{1}(\alpha )-z_{1}(\beta )+\tilde{f_1}(\alpha )-\tilde{f_1}(\beta ))(\frac{\partial _{\alpha }^{6}z_{\mu }(\alpha )}{1+ic'(\alpha )\gamma t}-\frac{\partial _{\beta }^{6}z_{\mu }(\beta )}{1+ic'(\beta )\gamma t})(1+ic'(\beta )\gamma t)}{\cosh (z_{2}(\alpha )-z_{2}(\beta )+\tilde{f_1}(\alpha )-\tilde{f_1}(\beta ))-\cos (z_{1}(\alpha )-z_{1}(\beta )+\tilde{f_1}(\alpha )-\tilde{f_1}(\beta ))}\text {d}\beta \\&\quad +\lambda (\alpha )\int _{-\pi }^{\pi } \frac{\sin (z_{1}(\alpha )-z_{1}(\beta )+\tilde{f_1}(\alpha )-\tilde{f_1}(\beta ))((\partial _{\alpha }^6\tilde{f_{\mu }})(\alpha )-(\partial _{\beta }^6\tilde{f_{\mu }})(\beta ))(1+ic'(\beta )\gamma t)}{\cosh (z_{2}(\alpha )-z_{2}(\beta )+\tilde{f_1}(\alpha )-\tilde{f_1}(\beta ))-\cos (z_{1}(\alpha )-z_{1}(\beta )+\tilde{f_1}(\alpha )-\tilde{f_1}(\beta ))}\text {d}\beta \\&\quad +\sum _i O^i=T_1(z)+T_2(z)+T_3(z)+\sum _i O^i. \end{aligned}$$

Here $O^i$ terms contain at most 5th derivative on both *z* and $\tilde{f}$.

Before we show the explicit form of $O^i$, we introduce some notations. Let

5.2 $$\begin{aligned} V^{k}_g(\alpha )&=(g_1(\alpha ,t), g_2(\alpha ,t), \partial _{\alpha }g_1(\alpha ,t),\partial _{\alpha }g_2(\alpha ,t), ...,\partial _{\alpha }^{k}g_1(\alpha ,t),\partial _{\alpha }^{k}g_2(\alpha ,t)), \nonumber \\ \tilde{V}^k_{g}(\alpha )&=\left( \frac{g_1(\alpha ,t)}{1+ic'(\alpha )\gamma t},\frac{g_2(\alpha ,t)}{1+ic'(\alpha )\gamma t},...,\partial _{\alpha }^k g_1(\alpha ,t)\partial _{\alpha }^{5}\left( \frac{1}{1+ic'(\alpha )\gamma t}\right) ,\right. \nonumber \\&\quad \left. \partial _{\alpha }^k g_2(\alpha ,t)\partial _{\alpha }^{5}\left( \frac{1}{1+ic'(\alpha )\gamma t}\right) \right) . \end{aligned}$$

$V_{g,i}^k(\alpha )$ is the ith component in $V_{g}^k(\alpha )$ and $\tilde{V}_{g,i}^k(\alpha )$ the ith component in $\tilde{V}_{g}^k(\alpha )$.

When we write $X_i(\alpha ,t)$, we mean

5.3 $$\begin{aligned} X_i(\alpha ,t)=\partial _{\alpha }^{l_i}\left( \frac{1}{1+ic'(\alpha )\gamma t}\right) , \end{aligned}$$

with $$l_i\le 5$$.

A function $K_{-\sigma }^{j}(A,B)$, $K_{-\sigma }^{j}(A,B,C)$ is of $-\sigma$ type if, for *A*, *B*, *C* in $R^n$, it has the form

5.4 $$\begin{aligned}&c_j\frac{\sin (A_1+B_1)^{m_1}\cos (A_1+B_1)^{m_2}}{(\cosh (A_2+B_2)-\cos (A_1+B_1))^{m_0}}\nonumber \\&\quad \times (\sinh (A_2+B_2))^{m_3}(\cosh (A_2+B_2))^{m_4}\Pi _{j=1}^{m_5}(A_{\lambda _{j}})\Pi _{j=1}^{m_6}(B_{\lambda _{j,2}})\Pi _{j=1}^{m_7}(C_{\lambda _{j,3}}), \end{aligned}$$

with $m_1+m_3+m_5+m_6+m_7-2m_0\ge -\sigma$. $c_j$ is a constant.

We claim that we can write $O^{i}$ as following three types, by separating the highest order term in the derivative. Here we omit the dependency on $\gamma$ and *t*.

$$\begin{aligned} O^{1,i}&=\partial _{\alpha }^{b_i}\lambda (\alpha )\int _{-\pi }^{\pi }K_{-1}^{i}(V_z^{3}(\alpha )\\&\quad -V_z^{3}(\beta ),V_{\tilde{f}}^{3}(\alpha )-V^{3}_{\tilde{f}}(\beta ),\tilde{V}_{z}^3(\alpha )-\tilde{V}_{z}^3(\beta ))X_{i'}(\beta )(\tilde{z}^{3}(\alpha )-\tilde{z}^3(\beta ))\text {d}\beta , \end{aligned}$$

where $$\tilde{z}^3\in V_z^{3} \cup \tilde{V}_z^{3}\cup V_{\tilde{f}}^3$$, $$1\le b_i\le 5$$.

$$\begin{aligned} O^{2,i}&=\partial _{\alpha }^{b_i}\lambda (\alpha )\int _{-\pi }^{\pi }K_{-1}^{i}(V_z^{2}(\alpha )\\&\quad -V_z^{2}(\beta ),V_{\tilde{f}}^{2}(\alpha )-V^{2}_{\tilde{f}}(\beta ),\tilde{V}_{z}^2(\alpha )-\tilde{V}_{z}^2(\beta ))X_{i'}(\beta )(\tilde{z}^{5}(\alpha )-\tilde{z}^5(\beta ))\text {d}\beta , \end{aligned}$$

where $$\tilde{z}^{5}\in V_z^{5} \cup \tilde{V}_z^{5}\cup V_{\tilde{f}}^5$$, $$1\le b_i\le 5$$.

$$\begin{aligned} O^{3,i}=\partial _{\alpha }^{b_i}\left( \frac{ic(\alpha )\gamma }{1+ic^{'}(\alpha )\gamma t}\right) \partial _{\alpha }^{b_i'}z_{\mu }(\alpha ), \end{aligned}$$

where $$1\le b_i, b_i'\le 5$$.

Then we have the following lemmas:

#### Lemma 5.1

We have

$$\begin{aligned} \Vert O^i\Vert _{L^2[-\pi ,\pi ]}\lesssim C(\Vert z\Vert _{U}, \Vert z\Vert _{Arc}). \end{aligned}$$

#### Proof

Since $K_{-1}^{i}$ is of $-1$ type, we have

$$\begin{aligned}{} & {} \Vert K_{-1}^{i}(V_z^{3}(\alpha )-V_z^{3}(\beta ),V_{\tilde{f}}^{3}(\alpha )\\{} & {} \quad -V^{3}_{\tilde{f}}(\beta ),\tilde{V}_{z}^3(\alpha )-\tilde{V}_{z}^3(\beta ))(\alpha -\beta )\Vert _{C^0[-2\delta ,2\delta ]\times [-\pi ,\pi ]}\lesssim C(\Vert z\Vert _{U},\Vert z\Vert _{Arc}), \end{aligned}$$

we could use Lemma 11.4 to get the result for $O^{1,i}$. Moreover, we have

$$\begin{aligned}{} & {} \Vert K_{-1}^{i}(V_z^{2}(\alpha )-V_z^{2}(\beta ),V_{\tilde{f}}^{2}(\alpha )\\{} & {} \quad -V^{2}_{\tilde{f}}(\beta ),\tilde{V}_{z}^2(\alpha )-\tilde{V}_{z}^2(\beta ))(\alpha -\beta )\Vert _{C^1[-2\delta ,2\delta ]\times [-\pi ,\pi ]}\lesssim C(\Vert z\Vert _{U},\Vert z\Vert _{Arc}), \end{aligned}$$

we then use Lemma 11.2 to get the estimate for $O^{2,i}$. $O^{3,i}$ can be bounded easily. $\square$

#### Lemma 5.2

We have

$$\begin{aligned} \Vert T_3(z)\Vert _{L^2[-\pi ,\pi ]}\lesssim C(\Vert z\Vert _{U}, \Vert z\Vert _{Arc}). \end{aligned}$$

#### Proof

Let

5.5 $$\begin{aligned} K(z(\alpha )-z(\beta ),\tilde{f}(\alpha )-\tilde{f}(\beta ))=\frac{\sin (z_1(\alpha )-z_1(\beta )+\tilde{f}_1(\alpha )-\tilde{f}_1(\beta ))}{\cosh (z_2(\alpha )-z_2(\beta )+\tilde{f}_2(\alpha )-\tilde{f}_2(\beta ))-\cos (z_1(\alpha )-z_1(\beta )+\tilde{f}_1(\alpha )-\tilde{f}_1(\beta ))}. \end{aligned}$$

It is also of $K_{-1}$ type. We have

$$\begin{aligned} \Vert K(z(\alpha )-z(\beta ),\tilde{f}(\alpha )-\tilde{f}(\beta ))(\alpha -\beta )\Vert _{C^1[-2\delta ,2\delta ]\times [-\pi ,\pi ]}\lesssim C(\Vert z\Vert _{U},\Vert z\Vert _{Arc}). \end{aligned}$$

Then the result follows from Lemma 11.2. $\square$

Then we are left to deal with $T_1+T_2$.

By using the same notation as in Lemma 5.2, we have

$$\begin{aligned} T_2(z(\alpha ))&=\lambda (\alpha )p.v.\int _{-\pi }^{\pi } K(z(\alpha )-z(\beta ),\tilde{f}(\alpha )-\tilde{f}(\beta ))(1+ic'(\beta )\gamma t)\text {d}\beta \frac{\partial _{\alpha }^{6}z_{\mu }(\alpha )}{(1+ic'(\alpha )\gamma t)}\\&\quad -\lambda (\alpha )p.v.\int _{-\pi }^{\pi } K(z(\alpha )-z(\beta ),\tilde{f}(\alpha )-\tilde{f}(\beta ))\partial _{\beta }^{6}z_{\mu }(\beta )\text {d}\beta \\&=T_{2,1}(z)(\alpha )+T_{2,2}(z)(\alpha ). \end{aligned}$$

Moreover, we could further split the $T_{2,2}$ and have

$$\begin{aligned} T_{2,2}(z)(\alpha )&= -\lambda (\alpha )p.v.\int _{-\pi }^{\pi } K(z(\alpha )-z(\beta ),\tilde{f}(\alpha )-\tilde{f}(\beta ))\partial _{\beta }^{6}z_{\mu }(\beta )\text {d}\beta \\&=-\lambda (\alpha )\lim _{\beta \rightarrow \alpha }\left( K(z(\alpha )-z(\beta ),\tilde{f}(\alpha )-\tilde{f}(\beta )) \tan \left( \frac{\alpha -\beta }{2}\right) \right) p.v.\\&\quad \int _{-\pi }^{\pi }\cot \left( \frac{\alpha -\beta }{2}\right) \partial _{\beta }^{6}z(\beta )\text {d}\beta \\&\quad -\lambda (\alpha )\int _{-\pi }^{\pi }\left( K(z(\alpha )-z(\beta ),\tilde{f}(\alpha ) -\tilde{f}(\beta ))\tan \left( \frac{\alpha -\beta }{2}\right) \right. \\&\quad \left. -\lim _{\beta \rightarrow \alpha }\left( K(z(\alpha )-z(\beta ), \tilde{f}(\alpha )-\tilde{f}(\beta ))\tan \left( \frac{\alpha -\beta }{2}\right) \right) \right) \\&\quad \cot \left( \frac{\alpha -\beta }{2}\right) \partial _{\beta }^{6}z(\beta )\text {d}\beta \\&=T_{2,2,1}(z)+T_{2,2,2}(z). \end{aligned}$$

Since $K(z(\alpha )-z(\beta ),\tilde{f}(\alpha )-\tilde{f}(\beta ))$ is of $-1$ type, we have

5.6 $$\begin{aligned}&\Vert K(z(\alpha )-z(\beta ),\tilde{f}(\alpha )-\tilde{f}(\beta )) \tan \left( \frac{\alpha -\beta }{2}\right) \Vert _{C^3_{\alpha ,\beta }([-2\delta ,2\delta ]\times [-\pi ,\pi ])}\nonumber \\&\quad \lesssim C(\Vert z\Vert _{Arc}+\Vert z\Vert _{U}). \end{aligned}$$

Let

$$\begin{aligned}&\tilde{K}(z(\alpha )-z(\beta ),\tilde{f}(\alpha )-\tilde{f}(\beta ))\\&\quad =\frac{K(z(\alpha )-z(\beta ),\tilde{f}(\alpha )-\tilde{f}(\beta )) \tan \left( \frac{\alpha -\beta }{2}\right) -\lim _{\beta \rightarrow \alpha }\left( K(z(\alpha ) -z(\beta ),\tilde{f}(\alpha )-\tilde{f}(\beta ))\tan \left( \frac{\alpha -\beta }{2}\right) \right) }{\tan \left( \frac{\alpha -\beta }{2}\right) }. \end{aligned}$$

Then

$$\begin{aligned}&\Vert \tilde{K}(z(\alpha )-z(\beta ),\tilde{f}(\alpha )-\tilde{f}(\beta ))\Vert _{C^2_{\alpha ,\beta }([-2\delta ,2\delta ]\times [-\pi ,\pi ])}\\&\quad \lesssim C(\Vert z\Vert _{Arc}+\Vert z\Vert _{U}). \end{aligned}$$

We can do the integration by parts in $T_{2,2,2}(z)$ to get that

$$\begin{aligned} T_{2,2,2}(z)(\alpha )&=-\lambda (\alpha )\int _{-\pi }^{\pi }\tilde{K}(z(\alpha )-z(\beta ),\tilde{f}(\alpha )-\tilde{f}(\beta ))\partial _{\beta }^{6}z(\beta )\text {d}\beta \\&=\lambda (\alpha )\int _{-\pi }^{\pi }\partial _{\beta }\tilde{K}(z(\alpha )-z(\beta ),\tilde{f}(\alpha )-\tilde{f}(\beta ))\partial _{\beta }^{5}z(\beta )\text {d}\beta . \end{aligned}$$

Therefore we have

$$\begin{aligned} \Vert T_{2,2,2}(z)(\alpha )\Vert _{L^2[-\pi ,\pi ]}\lesssim C(\Vert z\Vert _{Arc}+\Vert z\Vert _{U})\Vert \partial _{\alpha }^{5}z(\alpha )\Vert _{L^2[-\pi ,\pi ]}. \end{aligned}$$

In conclusion, we have

5.7 $$\begin{aligned} \frac{\text {d}\partial _{\alpha }^{5}z_{\mu }(\alpha ,\gamma ,t)}{dt}&=T_1(z)+T_{2,1}(z)+T_{2,2,1}(z)+(T_{2,2,2}(z)+T_3(z)+\sum _{i}O^{i}(z))\nonumber \\&=\left( \frac{ic(\alpha )\gamma }{1+ic^{'}(\alpha )\gamma t} +\frac{\lambda (\alpha )}{1+ic'(\alpha )\gamma t}p.v.\right. \nonumber \\&\quad \left. \int _{-\pi }^{\pi } K(z(\alpha )-z(\beta ), \tilde{f}(\alpha )-\tilde{f}(\beta ))(1+ic'(\beta )\gamma t)\text {d}\beta \right) \partial _{\alpha }^{6}z_{\mu }(\alpha )\nonumber \\&\quad -\lambda (\alpha )\lim _{\beta \rightarrow \alpha }\left( K(z(\alpha )-z(\beta ),\tilde{f}(\alpha ) -\tilde{f}(\beta ))\tan \left( \frac{\alpha -\beta }{2}\right) \right) 2\pi \Lambda (\partial _{\alpha }^5z)(\alpha )\nonumber \\&\quad +(T_{2,2,2}(z)+T_3(z)+\sum _{i}O^{i}(z)), \end{aligned}$$

where $\Lambda$ is $(-\Delta )^{\frac{1}{2}}$ on the Torus $\mathbb {T}$ of length $2\pi$ and

5.8 $$\begin{aligned} \left\| (T_{2,2,2}(z)+T_3(z)+\sum _{i}O^{i})\right\| _{L^2[-\pi ,\pi ]}\lesssim C(\Vert z\Vert _{Arc}+\Vert z\Vert _{U}). \end{aligned}$$

Then we have

5.9 $$\begin{aligned}&\frac{d}{dt}\Vert \partial _{\alpha }^5z(\alpha ,\gamma ,t)\Vert _{L^2_{\alpha }[-\pi ,\pi ]}^2=2\Re \int _{-\pi }^{\pi }\partial _{\alpha }^5z(\alpha ,\gamma ,t)\cdot \overline{\partial _{\alpha }^5\frac{d}{dt}z(\alpha ,\gamma ,t)}\text {d}\alpha \nonumber \\&\quad =\sum _{\mu =1,2}(\underbrace{2\Re \int _{-\pi }^{\pi }\partial _{\alpha }^5z_{\mu }(\alpha ) \overline{\left( \frac{ic(\alpha )\gamma }{1+ic^{'}(\alpha )\gamma t} +\frac{\lambda (\alpha )}{1+ic'(\alpha )\gamma t}p.v.\int _{-\pi }^{\pi } K(z(\alpha )-z(\beta ),\tilde{f}(\alpha ) -\tilde{f}(\beta ))(1+ic'(\beta )\gamma t)\text {d}\beta \right) }}_{\text {main term}}\nonumber \\&\qquad \underbrace{\overline{\cdot \partial _{\alpha }^{6}z_{\mu } (\alpha )}\text {d}\alpha }_{\text {main term}}\nonumber \\&\qquad \underbrace{-2\Re \int _{-\pi }^{\pi }\partial _{\alpha }^5z_{\mu }(\alpha ) \overline{\lambda (\alpha )\lim _{\beta \rightarrow \alpha }\left( K(z(\alpha )-z(\beta ),\tilde{f}(\alpha ) -\tilde{f}(\beta ))\tan \left( \frac{\alpha -\beta }{2}\right) \right) 2\pi \Lambda (\partial _{\alpha }^5z_{\mu }) (\alpha )}\text {d}\alpha }_{\text {main term}})\nonumber \\&+B.T, \end{aligned}$$

where $B.T.\le C(\Vert z\Vert _{Arc}+\Vert z\Vert _{U}).$ Next we show a lemma for controlling the main terms.

#### Lemma 5.3

If $L_1(\alpha )$, $L_2(\alpha )\in C^2(\mathbb {T})$, $-\Re L_1(\alpha )\ge |\Im L_2(\alpha )|$, $h\in H^1(\mathbb {T})$, then we have

$$\begin{aligned}&\Re \left( \int _{-\pi }^{\pi }h(\alpha )\overline{L_1(\alpha )(\Lambda h)(\alpha )}\text {d}\alpha +\int _{-\pi }^{\pi }h(\alpha )\overline{L_2(\alpha )\partial _{\alpha }h(\alpha )}\text {d}\alpha \right) \\&\quad \le C(\Vert L_1\Vert _{C^2(\mathbb {T})}+\Vert L_2\Vert _{C^2(\mathbb {T})})\Vert h(\alpha )\Vert _{L^2}^{2}. \end{aligned}$$

#### Proof

First, we have

$$\begin{aligned} I_2&=\Re \left( \int _{-\pi }^{\pi }h(\alpha )\overline{L_2(\alpha )\partial _{\alpha }h(\alpha )}\text {d}\alpha \right) \\&=\int _{-\pi }^{\pi }\Re L_2(\alpha )(\Re h(\alpha )\Re h'(\alpha )+\Im h(\alpha )\Im h'(\alpha ))\text {d}\alpha \\&\quad -\int _{-\pi }^{\pi }\Im L_2(\alpha )(\Re h(\alpha )\Im h'(\alpha )-\Im h(\alpha )\Re h'(\alpha ))\text {d}\alpha \\&=I_{2,1}+I_{2,s}. \end{aligned}$$

We could do the integration by parts to $I_{2,1}$ and have

$$\begin{aligned} |I_{2,1}|=\left| \frac{1}{2}\int _{-\pi }^{\pi }\frac{\text {d}\Re L_2(\alpha )}{\text {d}\alpha }((\Re h(\alpha ))^2 +(\Im h(\alpha ))^2)\text {d}\alpha \right| \lesssim C(\Vert L_2(\alpha )\Vert _{C^1(\mathbb {T})})\Vert h(\alpha )\Vert _{L^2}^2. \end{aligned}$$

Moreover,

$$\begin{aligned} I_1&=\Re \left( \int _{-\pi }^{\pi }h(\alpha )\overline{L_1(\alpha )(\Lambda h)(\alpha )}\text {d}\alpha \right) \\&=\int _{-\pi }^{\pi }\Re L_1(\alpha )(\Re h(\alpha )\Lambda \Re h(\alpha )+\Im h(\alpha )\Lambda \Im h(\alpha ))\text {d}\alpha \\&\quad -\int _{-\pi }^{\pi }\Im L_1(\alpha )(\Re h(\alpha )\Lambda \Im h(\alpha )-\Im h(\alpha )\Lambda \Re h(\alpha ))\text {d}\alpha \\&=I_{1,M}+I_{1,2}. \end{aligned}$$

We can still do the integration by parts to the $I_{1,2}$ and have

$$\begin{aligned}&\int _{-\pi }^{\pi }\Im L_1(\alpha )(\Re h(\alpha )\Lambda \Im h(\alpha )-\Im h(\alpha )\Lambda \Re h(\alpha ))\text {d}\alpha \\&\quad =\int _{-\pi }^{\pi }(\Lambda (\Im L_1\Re h)(\alpha )-\Im L_1(\alpha )\Lambda \Re h(\alpha ))\Im h(\alpha )\text {d}\alpha . \end{aligned}$$

Moreover, for any $g_1\in H^2(\mathbb {T})$, $g_2\in H^1(\mathbb {T})$, we have

$$\begin{aligned} \Vert \Lambda (g_1g_2)-g_1\Lambda g_2\Vert _{L^2(\mathbb {T})}\lesssim \Vert g_2\Vert _{L^2(\mathbb {T})}\Vert g_1\Vert _{H^2(\mathbb {T})}. \end{aligned}$$

Hence

$$\begin{aligned} I_{1,2}&\lesssim \Vert \Im L_1(\alpha )\Vert _{H^2(\mathbb {T})}\Vert \Im h(\alpha )\Vert _{L^2(\mathbb {T})}\Vert \Re h(\alpha )\Vert _{L^2(\mathbb {T})}\\&\lesssim \Vert \Im L_1(\alpha )\Vert _{H^2(\mathbb {T})}\Vert h(\alpha )\Vert _{L^2(\mathbb {T})}^2. \end{aligned}$$

Now we are left to control $I_{1,M}+I_{2,s}$. We have

$$\begin{aligned} I_{1,M}+I_{2,s}&= \int _{-\pi }^{\pi }\Re L_1(\alpha )(\Re h(\alpha )\Lambda \Re h(\alpha )+\Im h(\alpha )\Lambda \Im h(\alpha ))\text {d}\alpha \\&\quad +\int _{-\pi }^{\pi }\Im L_2(\alpha )(\Re h(\alpha )\Im h'(\alpha )-\Im h(\alpha )\Re h'(\alpha ))\text {d}\alpha . \end{aligned}$$

Now we use a lemma from [9, Section 2.4].

#### Lemma 5.4

Let *a*, *b* be real valued functions on $\mathbb {T}$, $a(\alpha )\ge |b(\alpha )|$ and satisfying $a,b \in C^2(\mathbb {T})$. Then we have

$$\begin{aligned} \Re \int _{\mathbb {T}}\overline{f(x)}(a(x)\Lambda f(x)+b(x)if'(x))dx\ge -C(\Vert a\Vert _{C^2(\mathbb {T})}+\Vert b\Vert _{C^2(\mathbb {T})})\int _{\mathbb {T}}|f(x)|^2dx. \end{aligned}$$

Then, from Lemma 5.4, we have

$$\begin{aligned} I_{1,M}+I_{2,s}\lesssim C(\Vert L_1\Vert _{C^2(\mathbb {T})}+\Vert L_2\Vert _{C^2(\mathbb {T})})\Vert h(\alpha )\Vert _{L^2}^{2}. \end{aligned}$$

Then we get the result. $\square$

Now let

5.10 $$\begin{aligned}&L_{z}^1(\alpha ,\gamma ,t)=-2\pi \lim _{\beta \rightarrow 0} \left( K(z(\alpha ,\gamma ,t)-z(\beta ,\gamma ,t),\tilde{f}(\alpha ,t)-\tilde{f}(\beta ,t))\tan \left( \frac{\alpha -\beta }{2}\right) \right) \nonumber \\&\quad =-2\pi \frac{\partial _{\alpha }z_1(\alpha ,\gamma ,t)+\partial _{\alpha }\tilde{f}_1(\alpha ,t)}{(\partial _{\alpha }z_1(\alpha ,\gamma ,t)+\partial _{\alpha }\tilde{f}_1(\alpha ,t))^2+(\partial _{\alpha }z_2(\alpha ,\gamma ,t)+\partial _{\alpha }\tilde{f}_2(\alpha ,t))^2}, \end{aligned}$$

and

5.11 $$\begin{aligned} L_{z}^2(\alpha ,\gamma ,t)&=\left( \frac{ic(\alpha )\gamma }{1+ic^{'}(\alpha )\gamma t} +\frac{1}{1+ic'(\alpha )\gamma t}p.v.\right. \nonumber \\&\quad \left. \int _{-\pi }^{\pi } K(z(\alpha ,\gamma ,t)-z(\beta ,\gamma ,t), \tilde{f}(\alpha ,t)-\tilde{f}(\beta ,t))(1+ic'(\beta )\gamma t)\text {d}\beta \right) . \end{aligned}$$

Since $\textrm{supp} c(\alpha )\subset \{\alpha | \lambda (\alpha )=1\}$, from (5.9), we have

$$\begin{aligned}&\frac{d}{dt}\Vert \partial _{\alpha }^5z(\alpha ,\gamma ,t)\Vert _{L^2_{\alpha }[-\pi ,\pi ]}^2\\&\quad =\sum _{\mu =1,2}\underbrace{2\Re \int _{-\pi }^{\pi }\partial _{\alpha }^5z_{\mu }(\alpha )\overline{\lambda (\alpha )L_{z}^2(\alpha )\partial _{\alpha }^{6}z_{\mu }(\alpha )}\text {d}\alpha }_{\text {main term}}\underbrace{+2\Re \int _{-\pi }^{\pi }\partial _{\alpha }^5z_{\mu }(\alpha )\overline{\lambda (\alpha )L_{z}^1(\alpha ) \Lambda (\partial _{\alpha }^5z_{\mu })(\alpha )}\text {d}\alpha }_{\text {main term}}\nonumber \\&\qquad +B.T. \end{aligned}$$

From Lemma 5.3, if $-\Re L_{z}^1(\alpha )\ge |\Im L_z^2(\alpha )|$ for $\alpha \in [-2\delta ,2\delta ]$, since $\textrm{supp} \lambda \subset [-2\delta ,2\delta ]$, we have

$$\begin{aligned}&\frac{d}{dt}\Vert \partial _{\alpha }^5z(\alpha ,\gamma ,t)\Vert _{L^2_{\alpha }[-\pi ,\pi ]}^2\lesssim B.T. \end{aligned}$$

Moreover, when $t=0$, $\alpha \in [-2\delta ,2\delta ]$, from (5.10), (5.11) and (5.5) we have

$$\begin{aligned} -\Re L_z^1(\alpha ,\gamma ,0)&=2\pi \frac{\partial _{\alpha }f_1^c(\alpha ,0)+\partial _{\alpha }\tilde{f}_1(\alpha ,0)}{(\partial _{\alpha }f_1^c(\alpha ,0)+\partial _{\alpha }\tilde{f}_1(\alpha ,0))^2+(\partial _{\alpha }f_2^c(\alpha ,0)+\partial _{\alpha }\tilde{f}_2(\alpha ,0))^2}\\&=2\pi \frac{\partial _{\alpha }f_1(\alpha ,0)}{(\partial _{\alpha }f_1(\alpha ,0))^2+(\partial _{\alpha }f_2(\alpha ,0))^2}, \end{aligned}$$

and

$$\begin{aligned} \Im L_z^2(\alpha ,0)&=ic(\alpha )\gamma +\Im (p.v.\int _{-\pi }^{\pi } K(f^c(\alpha ,0)-f^c(\beta ,0),\tilde{f}(\alpha ,0)-\tilde{f}(\beta ,0))\text {d}\beta )\\&=ic(\alpha )\gamma . \end{aligned}$$

From (3.1), we could choose $\delta _c$ in (4.1) to be sufficiently small and have

$$\begin{aligned} \inf _{\alpha \in [-2\delta ,2\delta ]}(-\Re L_z^1(\alpha ,\gamma ,0)-|\Im L_z^2(\alpha ,\gamma ,0)|)>0. \end{aligned}$$

Then let

$$\begin{aligned} \Vert z\Vert _{RT}(t)=\sup _{\alpha \in [-2\delta ,2\delta ]}\frac{1}{|\Re L_z^1(\alpha ,\gamma ,t)+|\Im L_z^2(\alpha ,\gamma ,t)||}. \end{aligned}$$

If $\Vert z\Vert _{RT}(t)< \infty$, we have

5.12 $$\begin{aligned} \frac{d}{dt}\Vert z\Vert _{U}^2\lesssim C(\Vert z\Vert _{U}+\Vert z\Vert _{Arc}). \end{aligned}$$

Therefore, we could let $\Vert z\Vert _{\tilde{U}}=\Vert z\Vert _{U}+\Vert z\Vert _{Arc}+\Vert z\Vert _{RT}$. From (5.12), and the following Lemma 5.5, we have

$$\begin{aligned} \frac{d}{dt}\Vert z\Vert _{\tilde{U}}^2\lesssim C(\Vert z\Vert _{\tilde{U}}). \end{aligned}$$

Then $\Vert z(\alpha ,\gamma ,t)\Vert _{\tilde{U}}$ is bounded for sufficiently small time $t_1$. We claim that the bound and the time can be chosen such that it holds for all $\gamma \in [-1,1]$.

#### Lemma 5.5

We have the following two estimates:

$$\begin{aligned} \frac{d}{dt}\Vert z\Vert _{Arc}\lesssim C(\Vert z\Vert _{\tilde{U}}), \end{aligned}$$

and

$$\begin{aligned} \frac{d}{dt}\Vert z\Vert _{RT}\lesssim C(\Vert z\Vert _{\tilde{U}}). \end{aligned}$$

#### Proof

For $\Vert z\Vert _{RT}$, we have

$$\begin{aligned}&\frac{d}{dt}\sup _{\alpha \in [-2\delta ,2\delta ]}\frac{1}{|\Re L_z^1(\alpha )+|\Im L_z^2(\alpha )||}\\&\quad \le \sup _{\alpha \in [-2\delta ,2\delta ]}\frac{1}{|\Re L_z^1(\alpha )+|\Im L_z^2(\alpha )||^2}\left( \left\| \frac{d}{dt}L_z^1(\alpha )\right\| _{L^{\infty }_{\alpha }[-2\delta ,2\delta ]}+\Vert \frac{d}{dt} L_z^2(\alpha )\Vert _{L^{\infty }_{\alpha }[-2\delta ,2\delta ]}\right) . \end{aligned}$$

From (5.10), we have

$$\begin{aligned}&\left\| \frac{d}{dt}L_z^1(\alpha )\right\| _{L^{\infty }_{\alpha }[-2\delta ,2\delta ]} \lesssim C(\Vert z\Vert _{\tilde{U}}) \left( \left\| \frac{d}{dt}z(\alpha ,\gamma ,t)\right\| _{C^1(\mathbb {T})} +\left\| \frac{d}{dt}\tilde{f}(\alpha ,t)\right\| _{C^1(\mathbb {T})}\right) \\&\quad \lesssim C(\Vert z\Vert _{\tilde{U}})(\Vert T(z)\Vert _{C^1(\mathbb {T})}+C). \end{aligned}$$

From (5.1), it is easy to get

$$\begin{aligned} \Vert T(z)\Vert _{C^1(\mathbb {T})}\lesssim C(\Vert z\Vert _{\tilde{U}}). \end{aligned}$$

Then

$$\begin{aligned}&\left\| \frac{d}{dt}L_z^1(\alpha )\right\| _{L^{\infty }_{\alpha }[-2\delta ,2\delta ]}\lesssim C(\Vert z\Vert _{\tilde{U}}). \end{aligned}$$

From (5.11), we have

$$\begin{aligned}&\left\| \frac{d}{dt}L_z^2(\alpha ,\gamma ,t)\right\| _{L^{\infty }_{\alpha }[-2\delta ,2\delta ]} \lesssim C+ C\Vert \frac{d}{dt}p.v.\int _{-\pi }^{\pi } K(z(\alpha )-z(\beta ),\tilde{f}(\alpha )\\&\qquad -\tilde{f}(\beta ))(1+ic'(\beta )\gamma t)\text {d}\beta \Vert _{L^{\infty }_{\alpha }[-2\delta ,2\delta ]}\\&\qquad +\Vert p.v.\int _{-\pi }^{\pi } K(z(\alpha )-z(\beta ),\tilde{f}(\alpha ) -\tilde{f}(\beta ))(1+ic'(\beta )\gamma t)\text {d}\beta \Vert _{L^{\infty }_{\alpha }[-2\delta ,2\delta ]}\\&\quad \le \Vert p.v.\int _{-\pi }^{\pi } \nabla _1 K(z(\alpha )-z(\beta ),\tilde{f} (\alpha )-\tilde{f}(\beta ))\cdot \\&\quad \left( \frac{dz}{dt}(\alpha )-\frac{dz}{dt}(\beta ) +\frac{\text {d}\tilde{f}}{dt}(\alpha )-\frac{\text {d}\tilde{f}}{dt}(\beta )\right) (1+ic'(\beta )\gamma t) \text {d}\beta \Vert _{L^{\infty }_{\alpha }[-2\delta ,2\delta ]}\\&\qquad +\Vert p.v.\int _{-\pi }^{\pi } K(z(\alpha )-z(\beta ),\tilde{f}(\alpha ) -\tilde{f}(\beta ))(ic'(\beta )\gamma )\text {d}\beta \Vert _{L^{\infty }_{\alpha }[-2\delta ,2\delta ]}\\&\qquad +\Vert p.v.\int _{-\pi }^{\pi } K(z(\alpha )-z(\beta ),\tilde{f}(\alpha ) -\tilde{f}(\beta ))(1+ic'(\beta )\gamma t)\text {d}\beta \Vert _{L^{\infty }_{\alpha }[-2\delta ,2\delta ]}+C\\&\quad =Term_{2,1}+Term_{2,2}+Term_{2,3}+C. \end{aligned}$$

From condition (5.6) and Lemma 11.1, we have

$$\begin{aligned} Term_{2,2}+Term_{2,3}\lesssim C(\Vert z\Vert _{\tilde{U}}). \end{aligned}$$

Moreover, $\nabla _1 K(z(\alpha )-z(\beta ),\tilde{f}(\alpha )-\tilde{f}(\beta ))$ is of $-2$ type, and

$$\begin{aligned}&\left\| \nabla _1 K(z(\alpha )-z(\beta ),\tilde{f}(\alpha )-\tilde{f}(\beta ))\cdot \left( \frac{dz}{dt}(\alpha ) -\frac{dz}{dt}(\beta )+\frac{\text {d}\tilde{f}}{dt}(\alpha ) -\frac{\text {d}\tilde{f}}{dt}(\beta )\right) (\alpha -\beta )\right\| _{C^1([-2\delta ,2\delta ]\times [-\pi ,\pi ])}\\&\quad \lesssim (\Vert T(z)\Vert _{C^2(\mathbb {T})}+C)C(\Vert z\Vert _{\tilde{U}}). \end{aligned}$$

From (5.1), it is easy to get

$$\begin{aligned} \Vert T(z)\Vert _{C^2(\mathbb {T})}\lesssim C(\Vert z\Vert _{\tilde{U}}). \end{aligned}$$

Then $$Term_{2,1}\lesssim C(\Vert z\Vert _{\tilde{U}})$$. Hence

$$\begin{aligned}&\left\| \frac{d}{dt}L_z^2(\alpha )\right\| _{L^{\infty }_{\alpha }[-2\delta ,2\delta ]}\lesssim C(\Vert z\Vert _{\tilde{U}}). \end{aligned}$$

Then we have the estimate

$$\begin{aligned}&\frac{d}{dt}\Vert z\Vert _{RT}=\frac{d}{dt}\sup _{\alpha \in [-2\delta ,2\delta ]}\frac{1}{|\Re L_z^1(\alpha )+|\Im L_z^2(\alpha )||}\lesssim C(\Vert z\Vert _{\tilde{U}}). \end{aligned}$$

Moreover, we have

$$\begin{aligned}&\frac{d}{dt}\Vert z\Vert _{Arc}=\frac{d}{dt}\sup _{\alpha \in [-2\delta ,2\delta ], \beta \in [-\pi ,\pi ]}\big |\frac{1}{(\frac{\cosh (z_{2}(\alpha )-z_{2}(\beta )+\tilde{f_1}(\alpha )-\tilde{f_1}(\beta ))-\cos (z_{1}(\alpha ,\gamma ,t)-z_{1}(\beta ,\gamma ,t)+\tilde{f_1}(\alpha )-\tilde{f_1}(\beta ))}{(\alpha -\beta )^2})}\big |\\&\quad \le \sup _{\alpha \in [-2\delta ,2\delta ],\beta \in [-\pi ,\pi ]} \big |\frac{1}{\left( \frac{\cosh (z_{2}(\alpha )-z_{2}(\beta )+\tilde{f_1}(\alpha )-\tilde{f_1}(\beta ))-\cos (z_{1}(\alpha ,\gamma ,t)-z_{1}(\beta ,\gamma ,t) +\tilde{f_1}(\alpha )-\tilde{f_1}(\beta ))}{(\alpha -\beta )^2}\right) }\big |^2\\&\quad \cdot \left\| \frac{d}{dt}\left( \frac{\cosh (z_{2}(\alpha )-z_{2}(\beta )+\tilde{f_1}(\alpha )-\tilde{f_1}(\beta ))-\cos (z_{1}(\alpha ,\gamma ,t)-z_{1}(\beta ,\gamma ,t)+\tilde{f_1}(\alpha ) -\tilde{f_1}(\beta ))}{(\alpha -\beta )^2}\right) \right\| _{L^{\infty }_{\alpha ,\beta }[-\pi ,\pi ]\times [-\pi ,\pi ]}\\&\quad \le \Vert z\Vert _{Arc}^2\\&\qquad \left( \left\| \frac{\sinh (z_{2}(\alpha )-z_{2}(\beta )+\tilde{f_2}(\alpha )-\tilde{f_2}(\beta ))}{\alpha -\beta }\frac{\frac{d}{dt}(z_2(\alpha )-z_2(\beta ))+\frac{d}{dt}(\tilde{f}_2(\alpha )-\tilde{f}_2(\beta ))}{(\alpha -\beta )}\right\| _{L^{\infty }_{\alpha ,\beta }[-\pi ,\pi ]\times [-\pi ,\pi ]}\right. \\&\qquad \left. +\left\| \frac{\sin (z_{1}(\alpha )-z_{1}(\beta )+\tilde{f_1}(\alpha )-\tilde{f_1}(\beta ))}{\alpha -\beta }\frac{\frac{d}{dt}(z_1(\alpha )-z_1(\beta ))+\frac{d}{dt}(\tilde{f}_1(\alpha )-\tilde{f}_2(\beta ))}{(\alpha -\beta )}\right\| _{L^{\infty }_{\alpha ,\beta }[-\pi ,\pi ]\times [-\pi ,\pi ]}\right) \\&\quad \lesssim \Vert z\Vert _{Arc}^2\Vert z\Vert _{U}(\Vert T(z)\Vert _{C^{1}_{\alpha }[-\pi ,\pi ]}+C)\\&\quad \lesssim C(\Vert z\Vert _{\tilde{U}}). \end{aligned}$$

We also introduce a corollary here to be used in a later section.

#### Corollary 5.6

For $g(\alpha )\in H^1(\mathbb {T})$, if $z\in H^5(\mathbb {T})$, $\Vert z\Vert _{Arc}< \infty$ and $-\Re L_z^1(\alpha )-|\Im L_z^2(\alpha )|>0$ when $\alpha \in [-2\delta ,2\delta ]$, $\gamma \in [-1,1]$, then we have

$$\begin{aligned}&\sup _{\gamma \in [-1,1]}(\int _{-\pi }^{\pi }g(\alpha )\overline{\lambda (\alpha )\int _{-\pi }^{\pi } K(z(\alpha )-z(\beta ),\tilde{f}(\alpha )-\tilde{f}(\beta ))\left( \frac{\partial _{\alpha }g(\alpha )}{1+ic'(\alpha )\gamma t}-\frac{\partial _{\alpha }g(\beta )}{1+ic'(\beta )\gamma t}\right) \text {d}\beta }\text {d}\alpha \\&\quad +\int _{-\pi }^{\pi }g(\alpha )\overline{\partial _{\alpha }g(\alpha )\frac{ic(\alpha )\gamma }{1+ic'(\alpha )\gamma t}}\text {d}\alpha )\lesssim \Vert g\Vert _{L^2}^2. \end{aligned}$$

$$\square $$

### Approximation for the picard theorem

Now we approximate the problem and have the following equations,

$$\begin{aligned}&\frac{d z^n(\alpha , \gamma ,t)}{dt}=\varphi _n*T^n(\varphi _n*z^n)=\varphi _n* \bigg (\frac{ic(\alpha )\gamma }{1+ic^{'}(\alpha )\gamma t}\partial _{\alpha }(\varphi _n*z^n)(\alpha )\bigg )\\&\quad +\varphi _n*\bigg (\lambda (\alpha )\int _{-\pi }^{\pi } K^n((\varphi _n*z^n)(\alpha ) -(\varphi _n*z^n)(\beta ),\tilde{f}(\alpha )-\tilde{f}(\beta ))\\&\quad \left( \frac{\partial _{\alpha }(\varphi _n*z_{\mu }^n)(\alpha )}{1+ic'(\alpha )\gamma t} -\frac{\partial _{\beta }(\varphi _n*z_{\mu }^n)(\beta )}{1+ic'(\beta )\gamma t}\right) (1+ic'(\beta )\gamma t)\text {d}\beta \bigg )\\&\quad +\varphi _n*\bigg (\lambda (\alpha )\int _{-\pi }^{\pi } K^n((\varphi _n*z^n)(\alpha )-(\varphi _n*z^n)(\beta ),\\&\quad \tilde{f}(\alpha )-\tilde{f}(\beta ))(\tilde{f}_{\mu }(\alpha )-\tilde{f}_{\mu }(\beta ))(1+ic'(\beta )\gamma t)d\beta \bigg ), \end{aligned}$$

where

$$\begin{aligned}&K^{n}((\varphi _n*z^n)(\alpha )-(\varphi _n*z^n)(\beta ),\tilde{f}(\alpha )-\tilde{f}(\beta ))\\&\quad =\sin (\Delta ((\varphi _n*z_{1}^n)(\alpha )+\tilde{f_1}(\alpha )))\\&\quad \cdot \frac{1}{|\cosh (\Delta ((\varphi _n*z_{2}^n)(\alpha )+\tilde{f_2}(\alpha ))) -\cos (\Delta ((\varphi _n*z_{1}^n)(\alpha )+\tilde{f_1}(\alpha )))|^2+\frac{1}{n} \sin \left( \frac{\alpha -\beta }{2}\right) ^2}\\&\quad \cdot \overline{\cosh (\Delta ((\varphi _n*z_{2}^n)(\alpha )+\tilde{f_2}(\alpha ))) -\cos (\Delta ((\varphi _n*z_{1}^n)(\alpha )+\tilde{f_1}(\alpha )))}, \end{aligned}$$

with

5.13 $$\begin{aligned} \Delta ((\varphi _n*z_{2}^n)(\alpha )+\tilde{f_2}(\alpha ))= & {} (\varphi _n*z_{2}^n)(\alpha )-(\varphi _n*z_{2}^n)(\beta )+\tilde{f_2}(\alpha )-\tilde{f_2}(\beta ),\nonumber \\ \end{aligned}$$

5.14 $$\begin{aligned} \Delta ((\varphi _n*z_{1}^n)(\alpha )+\tilde{f_1}(\alpha ))= & {} (\varphi _n*z_{1}^n)(\alpha )-(\varphi _n*z_{1}^n)(\beta )+\tilde{f_1}(\alpha )-\tilde{f_1}(\beta ),\nonumber \\ \end{aligned}$$

and initial value $z^n(\alpha , \gamma , 0)=\varphi _{n}*f(\alpha ,0)$. Here the convolution of $\varphi _n$ is the projection to the finite Fourier modes of $\alpha$.

By the Picard theorem, for any fixed $\gamma \in [-1,1]$, there exists solutions in $C^1([0,t_{n}], H^5_{\alpha }(\mathbb {T}))$. Moreover, by the structure of our approximation, we have $z^n=\varphi _{n}*z^n$, and for $1\le j\le 5$,

$$\begin{aligned}&\frac{d}{dt}\int _{-\pi }^{\pi }|\partial _{\alpha }^jz^n(\alpha ,\gamma ,t)|^2d\alpha \\&\quad =2\Re \int _{-\pi }^{\pi }\partial _{\alpha }^jz^n(\alpha ,\gamma ,t)\overline{\partial _{\alpha }^j(\varphi _n*T^n(\varphi _n*{z^n}))}d\alpha \\&\quad =2\Re \int _{-\pi }^{\pi }\partial _{\alpha }^j(\varphi _n*{z^n})(\alpha ,\gamma ,t)\overline{\partial _{\alpha }^j(T^n(\varphi _n*{z^n}))}d\alpha . \end{aligned}$$

Then we can do the energy estimate similar to that the previous section by letting

$$\begin{aligned} \Vert z^n\Vert _{\tilde{U}^n}=\Vert z^n\Vert _{H^5[-\pi ,\pi ]}+\Vert z^n\Vert _{RT^n}+\Vert z^n\Vert _{Arc^n}, \end{aligned}$$

where

$$\begin{aligned}&\Vert z^n\Vert _{Arc^n}=\sup _{\alpha \in [-2\delta ,2\delta ],\beta \in [-\pi ,\pi ]}\frac{|\cosh (\Delta ((\varphi _n*z_{2}^n)(\alpha )+\tilde{f_2}(\alpha )))-\cos (\Delta ((\varphi _n*z_{1}^n)(\alpha )+\tilde{f_1}(\alpha )))|(\alpha -\beta )^2}{|\cosh (\Delta ((\varphi _n*z_{2}^n)(\alpha )+\tilde{f_2}(\alpha )))-\cos (\Delta ((\varphi _n*z_{1}^n)(\alpha )+\tilde{f_1}(\alpha )))|^2+\frac{1}{n}\sin (\frac{\alpha -\beta }{2})^2}, \end{aligned}$$

with $\Delta ((\varphi _n*z_{2}^n)(\alpha )+\tilde{f_2}(\alpha ))$ and $\Delta ((\varphi _n*z_{1}^n)(\alpha )+\tilde{f_1}(\alpha ))$ from (5.13), (5.14), and letting

$$\begin{aligned} \Vert z^n\Vert _{RT^n}=\sup _{\alpha \in [-2\delta ,2\delta ]}\frac{1}{|\Re L_{z}^{1,n}(\alpha )+|\Im L_z^{2,n}(\alpha )||}, \end{aligned}$$

with

$$\begin{aligned} L_{z^n}^{1,n}(\alpha ,\gamma ,t)= & {} -2\pi \lim _{\beta \rightarrow 0}\left( K^{n}((\varphi _n*z^n)(\alpha )-(\varphi _n*z^n)(\beta ),\tilde{f}(\alpha ) -\tilde{f}(\beta ))\tan \left( \frac{\alpha -\beta }{2}\right) \right) ,\\ L_{z^n}^{2,n}(\alpha ,\gamma ,t)= & {} \left( \frac{ic(\alpha )\gamma }{1+ic^{'}(\alpha )\gamma t}+\frac{1}{1+ic'(\alpha )\gamma t}p.v.\right. \\{} & {} \quad \left. \int _{-\pi }^{\pi } K^n((\varphi _n*z^n)(\alpha )-(\varphi _n*z^n)(\beta ),\tilde{f}(\alpha )-\tilde{f}(\beta ))(1+ic'(\beta )\gamma t)d\beta \right) . \end{aligned}$$

Then we can use the similar energy estimate and the compactness argument to show there exist a solution

5.15 $$\begin{aligned} z(\alpha ,\gamma ,t)\in L^\infty ([0,t_1],H^5_{\alpha }(\mathbb {T})), \end{aligned}$$

satisfying

5.16 $$\begin{aligned} z(\alpha ,\gamma ,t)=\int _{0}^{t}T(z)d\tau +f^c(\alpha ,0), \end{aligned}$$

for sufficiently small time $t_1$. Moreover,

5.17 $$\begin{aligned} \Vert z\Vert _{Arc}<\infty , \end{aligned}$$

and

5.18 $$\begin{aligned} -\Re L_z^1(\alpha )-|\Im L_z^2(\alpha )|>0, \text { when } \alpha \in [-2\delta ,2\delta ]. \end{aligned}$$

Since the energy estimate has a bound for all $\gamma \in [-1,1]$, we have a existence time $t_1$ that holds for all $\gamma$.

Now we abuse the notation and write *T*(*z*) as $T(z(\alpha ,\gamma ,t),\gamma ,t)$. We have the following lemma:

#### Lemma 5.7

For any $g(\alpha ),h(\alpha )\in H^{j+1}(\mathbb {T})$, $j=3,4,$ $\Vert g\Vert _{Arc}< \infty$ and $\Vert h\Vert _{Arc}<\infty$, we have

5.19 $$\begin{aligned}&\Vert T(g(\alpha ),\gamma ,t)\Vert _{H^j(\mathbb {T})}\lesssim 1, \end{aligned}$$

5.20 $$\begin{aligned}&\Vert T(g(\alpha ),\gamma ,t)-T(h(\alpha ),\gamma ,t)\Vert _{H^j(\mathbb {T})}\lesssim \Vert g(\alpha )-g(\alpha )\Vert _{H^{j+1}(\mathbb {T})}, \end{aligned}$$

5.21 $$\begin{aligned}&\Vert T(g(\alpha ),\gamma ,t)-T(g(\alpha ),\gamma ,t')\Vert _{H^j(\mathbb {T})}\lesssim |t-t'|. \end{aligned}$$

#### Proof

We only show (5.19) and the left can be shown in the same way. From (5.1), we have

$$\begin{aligned}&T(g(\alpha ),\gamma ,t)=\frac{ic(\alpha )\gamma }{1+ic^{'}(\alpha )\gamma t}\partial _{\alpha }g(\alpha )\nonumber \\&\qquad +\lambda (\alpha )\int _{-\pi }^{\pi } K(g(\alpha )-g(\beta ),\tilde{f}(\alpha )-\tilde{f}(\beta )) \left( \frac{\partial _{\alpha }g(\alpha )}{1+ic'(\alpha )\gamma t}-\frac{\partial _{\beta }g(\beta )}{1+ic'(\beta )\gamma t}\right) (1+ic'(\beta )\gamma t)d\beta \nonumber \\&\qquad +\lambda (\alpha )\int _{-\pi }^{\pi } K(g(\alpha )-g(\beta ),\tilde{f}(\alpha )-\tilde{f}(\beta ))(\partial _{\alpha }\tilde{f}(\alpha )-\partial _{\beta }\tilde{f}(\beta ))(1+ic'(\beta )\gamma t)d\beta \\&\quad =T_{1}(g(\alpha ),\gamma ,t)+T_{2}(g(\alpha ),\gamma ,t)+T_{3}(g(\alpha ),\gamma ,t). \end{aligned}$$

It is trivial that $T_1$ satisfying the (5.19) since $c(\alpha )$ is sufficiently smooth.

Moreover, the $\partial _{\alpha }\tilde{f}\in H^5(\mathbb {T})$ and is more regular than $\frac{\partial _{\alpha }g(\alpha )}{1+ic'(\alpha )\gamma t}.$ Hence we only consider $T_2$. For $T_2$, we have

5.22 $$\begin{aligned} \Vert K(g(\alpha )-g(\beta ),\tilde{f}(\alpha )-\tilde{f}(\beta ))(\alpha -\beta )\Vert _{C^{j-2}([-2\delta ,2\delta ]\times \mathbb {T})}\lesssim 1. \end{aligned}$$

Then

$$\begin{aligned} \Vert T_2\Vert _{L^2(\mathbb {T})}\lesssim \Vert T_2\Vert _{L^\infty (\mathbb {T})}\lesssim 1. \end{aligned}$$

Moreover, we can use the notation from (5.4), (5.2), and get

$$\begin{aligned}&\partial _{\alpha }^{j}T_2(g)=\lambda (\alpha )\int _{-\pi }^{\pi } K(g(\alpha ) -g(\beta ),\tilde{f}(\alpha )-\tilde{f}(\beta ))\\&\qquad \left( \frac{\partial _{\alpha }^{j+1}g(\alpha )}{1+ic'(\alpha )\gamma t}-\frac{\partial _{\beta }^{j+1}g(\beta )}{1+ic'(\beta )\gamma t}\right) (1+ic'(\beta )\gamma t)d\beta \\&\qquad +\sum _{j'}\partial _{\alpha }^{b_{j'}}\lambda (\alpha ) \int _{-\pi }^{\pi }K_{-1}^{j'}(V_{g}^{\left[ \frac{j+1}{2}\right] }(\alpha )-V_{g}^{\left[ \frac{j+1}{2}\right] }(\beta ), V_{f}^{\left[ \frac{j+1}{2}\right] }(\alpha )-V_{f}^{\left[ \frac{j+1}{2}\right] }(\beta ),\\&\qquad \tilde{V}_{g}^{\left[ \frac{j+1}{2}\right] }(\alpha )-\tilde{V}_{g}^{\left[ \frac{j+1}{2}\right] }(\beta ))\\&\quad \cdot X_i(\beta )(\tilde{z}^{j}(\alpha )-\tilde{z}^{j}(\beta ))d\beta \\&\quad =Term_{2,1}+Term_{2,2}. \end{aligned}$$

Here $\tilde{z}^{j}\in V_g^{j} \cup \tilde{V}_g^{j}\cup V_{\tilde{f}}^j$. $[\frac{j+1}{2}]$ is the biggest integer less than $\frac{j+1}{2}$. Then from (5.22), we could use Lemma 11.2 to bound $Term_{2,1}$. Moreover, since $j+1-[\frac{j+1}{2}]\ge [\frac{j+1}{2}]\ge 2$. We have

$$\begin{aligned}&\left\| K_{-1}^{j'}(V_{g}^{\left[ \frac{j+1}{2}\right] }(\alpha )-V_{g}^{\left[ \frac{j+1}{2}\right] }(\beta ), V_{f}^{\left[ \frac{j+1}{2}\right] }(\alpha )-V_{f}^{\left[ \frac{j+1}{2}\right] }(\beta ),\right. \\&\qquad \left. \tilde{V}_{g}^{\left[ \frac{j+1}{2}\right] }(\alpha )-\tilde{V}_{g}^{\left[ \frac{j+1}{2}\right] }(\beta )) (\alpha -\beta )\right\| _{C^0([-2\delta ,2\delta ]\times [-\pi ,\pi ])}\\&\quad \lesssim C(\Vert g\Vert _{H^{j+1}}\Vert \tilde{f}\Vert _{H^{j+1}}). \end{aligned}$$

Then we could use Lemma 11.4 to bound $Term_{2,2}$. $\square$

Then from Lemma 5.7, (5.15) and (5.16), we have

5.23 $$\begin{aligned} z(\alpha ,\gamma ,t)\in L^\infty ([0,t_1],H^5_{\alpha }(\mathbb {T}))\cap C^0([0,t_1],H^4_{\alpha }(\mathbb {T}))\cap C^1([0,t_1],H^3_{\alpha }(\mathbb {T})). \end{aligned}$$

## The Uniqueness

In this section we show there exists sufficiently $0<t_2\le t_1$ such that for $0\le t\le t_2$, we have $z(\alpha ,0,t)=f^c(\alpha ,t)$.

Let $z^0(\alpha ,t)=z(\alpha ,0,t)$. From (5.1) and (5.5), we have

$$\begin{aligned} \frac{dz_{\mu }^0(\alpha ,t)}{dt}&=\lambda (\alpha )\int _{-\pi }^{\pi }K(z^0(\alpha )-z^{0}(\beta ),\tilde{f}(\alpha )-\tilde{f}(\beta ))(\partial _{\alpha }z^0_{\mu }(\alpha )-\partial _{\beta }z^0_{\mu }(\beta ))d\beta \\&\quad +\lambda (\alpha )\int _{-\pi }^{\pi }K(z^0(\alpha )-z^{0}(\beta ),\tilde{f}(\alpha )-\tilde{f}(\beta ))(\partial _{\alpha }\tilde{f}_{\mu }(\alpha )-\partial _{\beta }\tilde{f}_{\mu }(\beta ))d\beta . \end{aligned}$$

Moreover, from (3.2), we have

$$\begin{aligned} \frac{df^c_{\mu }(\alpha ,t)}{dt}&=\lambda (\alpha )\int _{-\pi }^{\pi }K(f^c(\alpha )-f^{c}(\beta ),\tilde{f}(\alpha )-\tilde{f}(\beta ))(\partial _{\alpha }f^c_{\mu }(\alpha )-\partial _{\beta }f^c_{\mu }(\beta ))d\beta \\&\quad +\lambda (\alpha )\int _{-\pi }^{\pi }K(f^c(\alpha )-f^{c}(\beta ),\tilde{f}(\alpha )-\tilde{f}(\beta ))(\partial _{\alpha }\tilde{f}_{\mu }(\alpha )-\partial _{\beta }\tilde{f}_{\mu }(\beta ))d\beta . \end{aligned}$$

Then we have the equation for the difference:

$$\begin{aligned}&\frac{d(z_{\mu }^0(\alpha ,t)-f^c(\alpha ,t))}{dt}=\lambda (\alpha )\int _{-\pi }^{\pi }K(z^0(\alpha )-z^{0}(\beta ),\tilde{f}(\alpha )-\tilde{f}(\beta ))(\partial _{\alpha }(z_{\mu }^0-f^c_{\mu })(\alpha )\\&\qquad -\partial _{\beta }(z_{\mu }^0-f^c_{\mu })(\beta ))d\beta \\&\qquad +\lambda (\alpha )\int _{-\pi }^{\pi }(K(z^0(\alpha )-z^{0}(\beta ),\tilde{f}(\alpha )-\tilde{f}(\beta ))-K(f^c(\alpha )-f^{c}(\beta ),\tilde{f}(\alpha )-\tilde{f}(\beta )))\\&\quad \cdot (\partial _{\alpha }\tilde{f}_{\mu }(\alpha )+\partial _{\alpha }f^c_{\mu }(\alpha )-\partial _{\beta }\tilde{f}_{\mu }(\beta )-\partial _{\beta }f^c_{\mu }(\beta ))d\beta \\&\quad =Term_{1}+Term_{2}. \end{aligned}$$

We first control $Term_2$, we have

6.1 $$\begin{aligned} Term_2&=\lambda (\alpha )\int _{-\pi }^{\pi }\int _{0}^1\frac{d}{d\tau }(K(f^c(\alpha )-f^{c}(\beta )\nonumber \\&\quad -\tau (f^c(\alpha )-z^0(\alpha )-(f^c(\beta )-z^0(\beta ))),\tilde{f}(\alpha )-\tilde{f}(\beta )))\nonumber \\&\cdot (\partial _{\alpha }\tilde{f}_{\mu }(\alpha )+\partial _{\alpha }f^c_{\mu }(\alpha )-\partial _{\beta }\tilde{f}_{\mu }(\beta )-\partial _{\beta }f^c_{\mu }(\beta ))d\tau d\beta \nonumber \\&=-\lambda (\alpha )\int _{-\pi }^{\pi }\int _{0}^1\nabla _1 K(f^c(\alpha )-f^{c}(\beta )-\tau (f^c(\alpha )-z^0(\alpha )\nonumber \\&\quad -(f^c(\beta )-z^0(\beta ))),\tilde{f}(\alpha )-\tilde{f}(\beta )))\nonumber \\&\cdot (f^c(\alpha )-z^0(\alpha )-(f^c(\beta )-z^0(\beta )))(\partial _{\alpha }\tilde{f}_{\mu }(\alpha )\nonumber \\&\quad +\partial _{\alpha }f^c_{\mu }(\alpha )-\partial _{\beta }\tilde{f}_{\mu }(\beta )-\partial _{\beta }f^c_{\mu }(\beta ))d\tau d\beta . \end{aligned}$$

Since the component of $\nabla K$ is of $-2$ type, we have

6.2 $$\begin{aligned}&\Vert \nabla _1 K(f^c(\alpha )-f^{c}(\beta )-\tau (f^c(\alpha )-z^0(\alpha )-(f^c(\beta )-z^0(\beta ))),\nonumber \\&\qquad \tilde{f}(\alpha )-\tilde{f}(\beta )))(\alpha -\beta )^2\Vert _{C^1_{\alpha ,\beta }([-2\delta ,2\delta ]\times [-\pi ,\pi ])}\nonumber \\&\quad \lesssim (\Vert f^c(\alpha )\Vert _{C^2[-\pi ,\pi ]}+\Vert z^0(\alpha )\Vert _{C^2[-\pi ,\pi ]}+\Vert \tilde{f}(\alpha )\Vert _{C^2[-\pi ,\pi ]})C(\Vert f^c-\tau (f^c-z^0)\Vert _{Arc}). \end{aligned}$$

When $t=0$, we have $z^0=f^c$, then

6.3 $$\begin{aligned} \Vert f^c-\tau (f^c-z^0)\Vert _{Arc}=\Vert f^c\Vert _{Arc}\lesssim 1. \end{aligned}$$

Moreover, we have the following lemma:

### Lemma 6.1

For $g,h\in C^1(\mathbb {T})$, $\Vert h\Vert _{Arc}< \infty$, there exists $\delta$ depending on $\Vert h\Vert _{Arc}$ and $\Vert h\Vert _{C^1(\mathbb {T})}$ such that when $\Vert g-h\Vert _{C^1(\mathbb {T})}\le \delta$, we have $\Vert g\Vert _{Arc}<\infty .$

### Proof

We have

$$\begin{aligned}&|\cosh (g_2(\alpha )-g_2(\beta )+\tilde{f}_2(\alpha )-\tilde{f}_2(\beta ))-\cosh (h_2(\alpha )-h_2(\beta )+\tilde{f}_2(\alpha )-\tilde{f}_2(\beta ))|\\&\quad \le |g_2(\alpha )-g_2(\beta )-(h_2(\alpha )-h_2(\beta ))|\int _{0}^{1}|\sinh ((1-\tau )(h_2(\alpha ) -h_2(\beta ))\\&\qquad +\tau (g_2(\alpha )-g_2(\beta ))+\tilde{f}_2(\alpha )-\tilde{f}_2(\beta ))|d\tau \\&\quad \le (\alpha -\beta )^2\Vert g-h\Vert _{C^1(T)}C(\Vert h\Vert _{C^1(T)}+\Vert g-h\Vert _{C^1(T)}), \end{aligned}$$

and

$$\begin{aligned}&|\cos (g_1(\alpha )-g_1(\beta )+\tilde{f}_1(\alpha )-\tilde{f}_1(\beta ))-\cos (h_1(\alpha )-h_1(\beta )+\tilde{f}_1(\alpha )-\tilde{f}_1(\beta ))|\\&\quad \le |g_1(\alpha )-g_1(\beta )-(h_1(\alpha )-h_1(\beta ))|\int _{0}^{1}|\sin ((1-\tau )(h_1(\alpha )-h_1(\beta ))\\&\quad +\tau (g_1(\alpha )-g_1(\beta ))+\tilde{f}_1(\alpha )-\tilde{f}_1(\beta ))|d\tau \\&\quad \le (\alpha -\beta )^2\Vert g-h\Vert _{C^1(T)}C(\Vert h\Vert _{C^1(T)}+\Vert g-h\Vert _{C^1(T)}). \end{aligned}$$

Since

$$\begin{aligned}{} & {} |\cosh (h_2(\alpha )-h_2(\beta )+\tilde{f}_2(\alpha )-\tilde{f}_2(\beta ))-\cos (h_1(\alpha )-h_1(\beta )+\tilde{f}_1(\alpha )-\tilde{f}_1(\beta ))|\\{} & {} \quad \ge \Vert h\Vert _{Arc}|\alpha -\beta |^2, \end{aligned}$$

we have

$$\begin{aligned}&|\cosh (g_2(\alpha )-g_2(\beta )+\tilde{f}_2(\alpha )-\tilde{f}_2(\beta ))-\cos (g_1(\alpha )-g_1(\beta )+\tilde{f}_1(\alpha )-\tilde{f}_1(\beta ))|\\&\quad \ge (\Vert h\Vert _{Arc}-\Vert g-h\Vert _{C^1(T)}C(\Vert h\Vert _{C^1(T)}+\Vert g-h\Vert _{C^1(T)}))|\alpha -\beta |^2. \end{aligned}$$

Then we have the result. $\square$

Since we have $z^0(\alpha ,t)\in C^{1}([0,t_1],H^3(\mathbb {T}))$, $f^c(\alpha ,t)\in C^{1}([0,t_1],H^6(\mathbb {T}))$, then from (6.3), and Lemma 6.1, there exists $t_2$, satisfying $0\le t_2\le t_1$, such that for $0\le t\le t_2$,

6.4 $$\begin{aligned} \Vert f^c-\tau (f^c-z^0)\Vert _{Arc}\lesssim 1. \end{aligned}$$

Then from corollary 11.3, (6.1),(6.2), and (6.4), we have

$$\begin{aligned} \Vert Term_2\Vert ^2_{L^2(\mathbb {T})}\le \Vert z^0-f^c\Vert ^2_{L^2(\mathbb {T})}. \end{aligned}$$

Then

$$\begin{aligned}&\frac{d}{dt}\int _{-\pi }^{\pi }|z^0(\alpha ,t)-f^c(\alpha ,t)|^2d\alpha \\&\quad =2\Re \int _{-\pi }^{\pi }(z^0(\alpha ,t)-f^c(\alpha ,t))\overline{\frac{d}{dt}(z^0(\alpha ,t)-f^c(\alpha ,t))}d\alpha \\&\quad =\sum _{\mu =1,2}2\Re \int _{-\pi }^{\pi }(z^0_{\mu }(\alpha ,t)-f^c_{\mu }(\alpha ,t))\overline{\lambda (\alpha )\int _{-\pi }^{\pi }K(z^0(\alpha )-z^{0}(\beta ),\tilde{f}(\alpha )-\tilde{f}(\beta ))(\partial _{\alpha }(z_{\mu }^0-f^c_{\mu })(\alpha )-\partial _{\beta }(z_{\mu }^0-f^c_{\mu })(\beta ))d\beta }d\alpha \\&\qquad +B.T^0, \end{aligned}$$

where $B.T^0\lesssim \Vert z^0(\alpha ,t)-f^c(\alpha ,t)\Vert _{L^2[-\pi ,\pi ]}^2$. Then from corollary 5.6 when $\gamma =0$, conditions (5.18), (5.15), (5.17), we have

$$\begin{aligned} \frac{d}{dt}\int _{-\pi }^{\pi }|z^0(\alpha ,t)-f^c(\alpha ,t)|^2d\alpha \lesssim \Vert z^0(\alpha ,t)-f^c(\alpha ,t)\Vert _{L^2(\mathbb {T})}^2. \end{aligned}$$

Moreover, we have $z^0(\alpha ,0)=f^c(\alpha ,0)$. Therefore we have

6.5 $$\begin{aligned} z^0(\alpha ,t)=f^c(\alpha ,t), \end{aligned}$$

for $$0\le t\le t_2$$.

## The Continuity of z with Respect to $\gamma$

We first show $\Vert z(\alpha , \gamma , t)-z(\alpha ,\gamma ',t)\Vert _{H^3(\alpha )}\lesssim |\gamma -\gamma '|$.

For the sake of simplicity, we further shrink the time $t_1$ to $\tilde{t}_1$ such that for all $0\le t\le \tilde{t}_1$, $\gamma ,\gamma '\in [-1,1]$, $\tau \in [0,1]$, we have $\Vert \tau z(\alpha ,\gamma ,t)+(1-\tau )z(\alpha ,\gamma ',t)-\tau z(\alpha ,\gamma ,0)-(1-\tau )z(\alpha ,\gamma ',0)\Vert _{C^1(\mathbb {T})}=\Vert \tau z(\alpha ,\gamma ,t)+(1-\tau )z(\alpha ,\gamma ',t)-f^c(\alpha ,0)\Vert _{C^1(\mathbb {T})}$ is sufficiently small. Then from Lemma 6.1, we have

$$\begin{aligned} \Vert \tau z(\alpha ,\gamma ,t)+(1-\tau )z(\alpha ,\gamma ',t)\Vert _{Arc}<\infty . \end{aligned}$$

This is not necessary but helps to simplify our estimate in this section.

Now we estimate the difference, we have

7.1 $$\begin{aligned}&\frac{dz(\alpha ,\gamma )}{dt}-\frac{dz(\alpha ,\gamma ')}{dt}=T(z(\alpha ,\gamma ,t),\gamma ,t)-T(z(\alpha ,\gamma ',t),\gamma ',t)\nonumber \\&\quad =(T(z(\alpha ,\gamma ,t),\gamma ,t)-T(z(\alpha ,\gamma ',t),\gamma ,t)) +\left( \int _{\gamma }^{\gamma '}(\partial _{\eta }T)(z(\alpha ,\gamma ',t),\eta ,t)d\eta \right) \nonumber \\&\quad =Term_1+Term_2. \end{aligned}$$

For $Term_2$, we have

7.2 $$\begin{aligned}&(\partial _{\gamma }T)(z(\alpha ),\gamma ,t)=\frac{d}{d\gamma } \left( \frac{ic(\alpha )\gamma }{1+ic'(\alpha )\gamma t}\right) \partial _{\alpha }z(\alpha )\nonumber \\&\qquad +\lambda (\alpha )\int _{-\pi }^{\pi }K(z(\alpha )-z(\beta ),\tilde{f}(\alpha )-\tilde{f}(\beta ))\frac{d}{d\gamma }(\frac{1+ic'(\beta )\gamma t}{1+ic'(\alpha )\gamma t}-1)d\beta \partial _{\alpha }z(\alpha )\nonumber \\&\qquad +\lambda (\alpha )\int _{-\pi }^{\pi }K(z(\alpha )-z(\beta ),\tilde{f}(\alpha )-\tilde{f}(\beta ))(\partial _{\alpha }\tilde{f}(\alpha )-\partial _{\beta }\tilde{f}(\beta ))ic'(\beta )t d\beta \nonumber \\&\quad =Term_{2,1}+Term_{2,2}+Term_{2,3}. \end{aligned}$$

Since $z(\alpha ,\gamma ,t)\in L^{\infty }_{t}([0,t_0],H^5(\mathbb {T}))$, we have

$$\begin{aligned} \Vert Term_{2,1}\Vert _{H^3(\mathbb {T})}\lesssim 1. \end{aligned}$$

Moreover,

$$\begin{aligned} Term_{2,2}&=\lambda (\alpha )\int _{-\pi }^{\pi }K(z(\alpha )-z(\beta ),\tilde{f}(\alpha ) -\tilde{f}(\beta ))\\&\quad \left[ \frac{(ic(\beta )-ic(\alpha ))t}{1+ic'(\alpha )\gamma t}-\frac{(ic(\beta )-ic(\alpha ))\gamma tic'(\alpha )t}{(1+c'(\alpha )\gamma t)^2}\right] d\beta \partial _{\alpha }z(\alpha ). \end{aligned}$$

Since *K* is of $-1$ type, we have

7.3 $$\begin{aligned} K(z(\alpha )-z(\beta ),\tilde{f}(\alpha )-\tilde{f}(\beta ))(\alpha -\beta )\in C^3([-2\delta ,2\delta ]\times [-\pi ,\pi ]). \end{aligned}$$

Therefore $\Vert Term_{2,2}\Vert _{H^3(\mathbb {T})}\lesssim 1$. Moreover, $Term_{2,3}$ can be bounded in the similar way since $|\frac{\partial _{\alpha }\tilde{f}(\alpha )-\partial _{\beta }\tilde{f}(\beta )}{\alpha -\beta }|\in C^3([-2\delta ,2\delta ]\times [-\pi ,\pi ])$ and we get

$$\begin{aligned} \Vert Term_{2,3}\Vert _{H^3(\mathbb {T})}\lesssim 1. \end{aligned}$$

Then we have

7.4 $$\begin{aligned} \Vert (\partial _{\gamma }T)(z(\alpha ),\gamma ,t)\Vert _{H^3(\mathbb {T})}\lesssim 1, \end{aligned}$$

and

7.5 $$\begin{aligned} \Vert Term_2\Vert _{H^3(\mathbb {T})}\lesssim |\gamma -\gamma '|. \end{aligned}$$

For $Term_1$, notice that $\tilde{f}(\alpha +ic(\alpha )\gamma t,t)=\tilde{f}(\alpha ,t)$, we have

7.6 $$\begin{aligned} Term_1&=\frac{ic(\alpha )\gamma }{1+ic'(\alpha )\gamma t}\partial _{\alpha }(z(\alpha ,\gamma ) -z(\alpha ,\gamma '))\nonumber \\&\quad +\lambda (\alpha )\int _{-\pi }^{\pi }K(z(\alpha ,\gamma )-z(\beta ,\gamma ),\tilde{f}(\alpha ) -\tilde{f}(\beta ))\left( \frac{\partial _{\alpha }z(\alpha ,\gamma )-\partial _{\alpha }z(\alpha ,\gamma ')}{1+ic'(\alpha )\gamma t}\right. \nonumber \\&\quad \left. -\left( \frac{\partial _{\beta }z(\beta ,\gamma )-\partial _{\beta }z(\beta ,\gamma ')}{1+ic'(\beta )\gamma t}\right) \right) \nonumber \\&\quad \cdot (1+ic'(\beta )\gamma t)d\beta \nonumber \\&\quad +\lambda (\alpha )\int _{-\pi }^{\pi }(K(z(\alpha ,\gamma )-z(\beta ,\gamma ),\tilde{f}(\alpha ) -\tilde{f}(\beta ))-K(z(\alpha ,\gamma ')\nonumber \\&\quad -z(\beta ,\gamma '),\tilde{f}(\alpha )-\tilde{f}(\beta )))\nonumber \\&\quad \left( \frac{\partial _{\alpha }z(\alpha ,\gamma ')}{1+ic'(\alpha )\gamma t} -\frac{\partial _{\beta }z(\beta ,\gamma ')}{1+ic'(\beta )\gamma t}+\partial _{\alpha }\tilde{f}(\alpha ) -\partial _{\beta }\tilde{f}(\beta )\right) (1+ic'(\beta )\gamma t)d\beta \nonumber \\&=Term_{1,1}+Term_{1,2}+Term_{1,3}. \end{aligned}$$

It is easy to get that

7.7 $$\begin{aligned} \Vert Term_{1,1}\Vert _{L^2(T)}+\Vert Term_{1,2}\Vert _{L^2(T)}\lesssim \Vert z(\alpha ,\gamma )-z(\alpha ,\gamma ')\Vert _{H^3(\mathbb {T})}.\end{aligned}$$

Moreover,

7.8 $$\begin{aligned} Term_{1,3}&=\lambda (\alpha )\int _{-\pi }^{\pi }\int _{0}^{1}\nabla _1 K(\tau (z(\alpha ,\gamma )-z(\beta ,\gamma )) +(1-\tau )(z(\alpha ,\gamma ')-z(\beta ,\gamma ')),\nonumber \\&\quad \tilde{f}(\alpha )-\tilde{f}(\beta ))d\tau \nonumber \\&\cdot (z(\alpha ,\gamma )-z(\beta ,\gamma )-(z(\alpha ,\gamma ')-z(\beta ,\gamma ')))\nonumber \\&\cdot \left( \frac{\partial _{\alpha }z(\alpha ,\gamma ')}{1+ic'(\alpha )\gamma t} -\frac{\partial _{\beta }z(\beta ,\gamma ')}{1+ic'(\beta )\gamma t}+\partial _{\alpha }\tilde{f}(\alpha ) -\partial _{\beta }\tilde{f}(\beta )\right) (1+ic'(\beta )\gamma t) d\beta . \end{aligned}$$

Since the component of $$\nabla _1 K$$ is of $$-2 $$ type, we have

$$\begin{aligned}{} & {} \sup _{\tau }\Vert \nabla _1 K(\tau (z(\alpha ,\gamma )-z(\beta ,\gamma )+(1-\tau )(z(\alpha ,\gamma ')\\{} & {} \quad -z(\beta ,\gamma ')),\tilde{f}(\alpha )-\tilde{f}(\beta ))(\alpha -\beta )^2\Vert _{C^3([-2\delta ,2\delta ]\times [-\pi ,\pi ])}\lesssim 1, \end{aligned}$$

then we have

7.9 $$\begin{aligned} \Vert Term_{1,3}\Vert _{L^2(T)}\lesssim \Vert z(\alpha ,\gamma )-z(\alpha ,\gamma ')\Vert _{H^3(T)}. \end{aligned}$$

Now we control $\partial _{\alpha }^3 Term_1$. For $\partial _{\alpha }^{3}Term_{1,1}$, we have

7.10 $$\begin{aligned} \partial _{\alpha }^{3}Term_{1,1}&=\frac{ic(\alpha )\gamma }{1+ic'(\alpha )\gamma t}\partial _{\alpha }^4(z(\alpha ,\gamma )-z(\alpha ,\gamma '))\nonumber \\&\quad +\sum _{1\le j\le 3} C_{1,j}\partial _{\alpha }^{j}\left( \frac{ic(\alpha )\gamma }{1+ic'(\alpha )\gamma t}\right) \partial _{\alpha }^{4-j}(z(\alpha ,\gamma )-z(\alpha ,\gamma '))\nonumber \\&=Term_{1,1,M}^{3}+Term_{1,1,2}^3. \end{aligned}$$

Here $\Vert Term_{1,1,2}^3\Vert _{L^2(\mathbb {T})}\lesssim \Vert z(\alpha ,\gamma )-z(\alpha ,\gamma ')\Vert _{H^3(\mathbb {T})}$. For $\partial _{\alpha }^{3}Term_{1,2}$, from Lemma 11.6 and (7.3), we have

7.11 $$\begin{aligned}&\partial _{\alpha }^3 Term_{1,2}=\lambda (\alpha )\int _{-\pi }^{\pi }K(z(\alpha ,\gamma )-z(\alpha -\beta ,\gamma ),\tilde{f}(\alpha )-\tilde{f}(\alpha -\beta ))\nonumber \\&\quad \cdot \left( \partial _{\alpha }^{3}\left( \frac{\partial _{\alpha }(z(\alpha ,\gamma )-z(\alpha ,\gamma '))}{1+ic'(\alpha )\gamma t}\right) -\partial _{\alpha }^{3}\left( \frac{\partial _{\alpha }(z(\alpha -\beta ,\gamma )-z(\alpha -\beta ,\gamma '))}{1+ic'(\alpha -\beta )\gamma t}\right) \right) \nonumber \\&\quad (1+ic'(\alpha -\beta )\gamma t)d\beta +Term_{1,2,1}^3, \end{aligned}$$

where $\Vert Term_{1,2,1}^3\Vert _{L^2(\mathbb {T})}\lesssim \Vert z(\alpha ,\gamma )-z(\alpha ,\gamma ')\Vert _{H^3(\mathbb {T})}$. Moreover, we have

7.12 $$\begin{aligned}&\lambda (\alpha )\int _{-\pi }^{\pi }K(z(\alpha ,\gamma )-z(\alpha -\beta ,\gamma ),\tilde{f}(\alpha )-\tilde{f}(\alpha -\beta ))\nonumber \\&\quad \cdot \left( \partial _{\alpha }^{3}\left( \frac{\partial _{\alpha }(z(\alpha ,\gamma )-z(\alpha ,\gamma '))}{1+ic'(\alpha )\gamma t}\right) -\partial _{\alpha }^{3}(\frac{\partial _{\alpha }(z(\alpha -\beta ,\gamma ) -z(\alpha -\beta ,\gamma '))}{1+ic'(\alpha -\beta )\gamma t})\right) \nonumber \\&\quad (1+ic'(\alpha -\beta )\gamma t)d\beta \nonumber \\&\quad = \lambda (\alpha )\int _{-\pi }^{\pi }K(z(\alpha ,\gamma )-z(\alpha -\beta ,\gamma ),\tilde{f}(\alpha ) -\tilde{f}(\alpha -\beta ))\nonumber \\&\quad \cdot \left( \frac{\partial _{\alpha }^4(z(\alpha ,\gamma )-z(\alpha ,\gamma '))}{1+ic'(\alpha )\gamma t} -\frac{\partial _{\alpha }^4(z(\alpha -\beta ,\gamma )-z(\alpha -\beta ,\gamma '))}{1+ic'(\alpha -\beta )\gamma t}\right) )(1+ic'(\alpha -\beta )\gamma t)d\beta \nonumber \\&\qquad +\sum _{0\le j\le 2} C_j \lambda (\alpha )\int _{-\pi }^{\pi }K(z(\alpha ,\gamma ) -z(\alpha -\beta ,\gamma ),\tilde{f}(\alpha )-\tilde{f}(\alpha -\beta ))(1+ic'(\alpha -\beta )\gamma t)\nonumber \\&\quad \cdot \left( \partial _{\alpha }^{1+j}(z(\alpha ,\gamma )-z(\alpha ,\gamma ')) \partial _{\alpha }^{3-j}\left( \frac{1}{1+ic'(\alpha )\gamma t}\right) -\partial _{\alpha }^{1+j} (z(\alpha -\beta ,\gamma )\right. \nonumber \\&\qquad \left. -z(\alpha -\beta ,\gamma '))\partial _{\alpha }^{3-j} \left( \frac{1}{1+ic'(\alpha -\beta )\gamma t}\right) \right) d\beta \nonumber \\&\quad =Term_{1,2,M}^{3}+Term_{1,2,3}^{3}. \end{aligned}$$

Then from Lemma 11.2, we have

7.13 $$\begin{aligned} \Vert Term_{1,2,3}^{3}\Vert _{L^2(\mathbb {T})}\lesssim \Vert z(\alpha ,\gamma )-z(\alpha ,\gamma ')\Vert _{H^3(\mathbb {T})}. \end{aligned}$$

For $\partial _{\alpha }^3 Term_{1,3}$, we use equation (7.8). Since

$$\begin{aligned}&\Vert \nabla K(\tau (z(\alpha ,\gamma )-z(\beta ,\gamma ))+(1-\tau )(z(\alpha ,\gamma ')-z(\beta ,\gamma ')),\tilde{f}(\alpha )-\tilde{f}(\beta ))(\alpha -\beta )^2 \Vert _{C^3([-2\delta ,2\delta ]\times [-\pi ,\pi ])}\lesssim 1, \end{aligned}$$

and

$$\begin{aligned} \frac{\partial _{\alpha }z(\alpha ,\gamma ')}{1+ic'(\alpha )\gamma t}+\partial _{\alpha }\tilde{f}(\alpha )\in H^4(\mathbb {T}), \end{aligned}$$

from Lemma 11.7 we have

7.14 $$\begin{aligned} \Vert \partial _{\alpha }^3 Term_{1,3}\Vert _{L^2(\mathbb {T})}\lesssim \Vert z(\alpha ,\gamma )-z(\alpha ,\gamma ')\Vert _{H^3(\mathbb {T})}. \end{aligned}$$

In conclusion, from (7.7), (7.9), and (7.5), we have

$$\begin{aligned}&\frac{d}{dt}\int _{-\pi }^{\pi }|z(\alpha ,\gamma )-z(\alpha ,\gamma ')|^2d\alpha =2\Re \int _{-\pi }^{\pi } (z(\alpha ,\gamma )-z(\alpha ,\gamma '))\overline{Term_{1}+Term_{2}}d\alpha \\&\quad \lesssim |\gamma -\gamma '|^2+\Vert z(\alpha ,\gamma )-z(\alpha ,\gamma ')\Vert _{H^3(\mathbb {T})}^2. \end{aligned}$$

From (7.10), (7.11), (7.12), (7.13), (7.14) and (7.5), we have

$$\begin{aligned}&\frac{d}{dt}\int _{-\pi }^{\pi }|\partial _{\alpha }^3(z(\alpha ,\gamma )-z(\alpha ,\gamma '))|^2d\alpha \\&\quad =2\Re \int _{-\pi }^{\pi }\partial _{\alpha }^3(z(\alpha ,\gamma )-z(\alpha ,\gamma '))\cdot \overline{\partial _{\alpha }^3 Term_1+\partial _{\alpha }^3 Term_2}d\alpha \\&\quad =2\Re \int _{-\pi }^{\pi }\partial _{\alpha }^3(z(\alpha ,\gamma )-z(\alpha ,\gamma '))\cdot \overline{ Term_{1,1,M}^3+Term_{1,2,M}^3}d\alpha +B.T^0\\&\quad =\sum _{\mu =1,2}2\Re \int _{-\pi }^{\pi }\partial _{\alpha }^3(z_{\mu }(\alpha ,\gamma )-z_{\mu }(\alpha ,\gamma '))\overline{\frac{ic(\alpha )\gamma }{1+ic'(\alpha )\gamma t}\partial _{\alpha }^4(z_{\mu }(\alpha ,\gamma )-z(\alpha ,\gamma '))}d\alpha \\&\qquad +\sum _{\mu =1,2}2\Re \int _{-\pi }^{\pi }\partial _{\alpha }^3(z_{\mu }(\alpha ,\gamma )-z_{\mu }(\alpha ,\gamma '))\overline{\lambda (\alpha )\int _{-\pi }^{\pi }K(z(\alpha ,\gamma )-z(\alpha -\beta ,\gamma ),\tilde{f}(\alpha )-\tilde{f}(\alpha -\beta ))}\nonumber \\&\qquad \overline{\cdot \left( \frac{\partial _{\alpha }^4(z(\alpha ,\gamma )-z(\alpha ,\gamma '))}{1+ic'(\alpha )\gamma t}-\frac{\partial _{\alpha }^4(z(\alpha -\beta ,\gamma )-z(\alpha -\beta ,\gamma '))}{1+ic'(\alpha -\beta )\gamma t}\right) )(1+ic'(\alpha -\beta )\gamma t)d\beta }d\alpha \\&\qquad +B.T^0, \end{aligned}$$

where

$$\begin{aligned} |B.T^0|\lesssim \Vert z(\alpha ,\gamma )-z(\alpha ,\gamma ')\Vert _{H^3(\mathbb {T})}^2+|\gamma -\gamma '|^2. \end{aligned}$$

Then from corollary 5.6, we have

$$\begin{aligned}&\frac{d}{dt}\int _{-\pi }^{\pi }|\partial _{\alpha }^3(z(\alpha ,\gamma )-z(\alpha ,\gamma '))|^2d\alpha \lesssim \Vert z(\alpha ,\gamma )-z(\alpha ,\gamma ')\Vert _{H^3(\mathbb {T})}^2+|\gamma -\gamma '|^2. \end{aligned}$$

Moreover, the initial date $\Vert z(\alpha ,\gamma )-z(\alpha ,\gamma ')\Vert _{H^3(\mathbb {T})}^2|_{t=0}=0$. Therefore we have

7.15 $$\begin{aligned} \left\| \frac{z(\alpha ,\gamma )-z(\alpha ,\gamma ')}{\gamma -\gamma '}\right\| _{H^3(\mathbb {T})}\lesssim 1. \end{aligned}$$

## The Differentiability of z with Respect to $\gamma$

Now we show the differentiability. We define a new function $w(\alpha ,\gamma ,t)$. It satisfies the equation that $\frac{dz}{d\gamma }$ would satisfy if it is differentiable.

Let *w* be the solution of the equation

8.1 $$\begin{aligned} \frac{dw(\alpha ,\gamma ,t)}{dt}=\tilde{T}(w)=D_zT(z(\alpha ,\gamma ,t),\gamma ,t)[w]+\partial _{\gamma }T(\alpha ,\gamma ,t), \end{aligned}$$

with initial value $w(\alpha ,\gamma ,0)=0$. Here $D_z T(z(\alpha ,\gamma ,t),\gamma ,t)[w]$ is the Gateaux derivative.

As in the existence of $z(\alpha ,\gamma ,t)$, we first show the energy estimate. First, from (7.4), we have

$$\begin{aligned} \Vert (\partial _{\gamma }T)(z(\alpha ),\gamma ,t)\Vert _{H^3(\mathbb {T})}\lesssim 1. \end{aligned}$$

Moreover,

8.2 $$\begin{aligned}&D_zT(z(\alpha ,\gamma ,t),\gamma ,t)[w]=\frac{ic(\alpha )\gamma }{1+ic^{'}(\alpha )\gamma t}\partial _{\alpha }w(\alpha ,\gamma )\nonumber \\&\quad +\lambda (\alpha )\int _{-\pi }^{\pi } K(z(\alpha ,\gamma )-z(\beta ,\gamma ),\tilde{f}(\alpha ) -\tilde{f}(\beta ))\left( \frac{\partial _{\alpha }w(\alpha ,\gamma )}{1+ic'(\alpha )\gamma t}-\frac{\partial _{\beta }w(\beta ,\gamma )}{1+ic'(\beta )\gamma t}\right) \nonumber \\&\quad (1+ic'(\beta )\gamma t)d\beta \nonumber \\&\quad +\lambda (\alpha )\int _{-\pi }^{\pi } (\nabla _1 K(z(\alpha ,\gamma )-z(\beta ,\gamma ),\tilde{f}(\alpha )-\tilde{f}(\beta ))\cdot (w(\alpha ,\gamma )-w(\beta ,\gamma )))\nonumber \\&\quad \cdot \left( \partial _{\alpha }\tilde{f}(\alpha )-\partial _{\beta }\tilde{f}(\beta )+\frac{\partial _{\alpha }z(\alpha ,\gamma )}{1+ic'(\alpha )\gamma t}-\frac{\partial _{\beta }z(\beta ,\gamma )}{1+ic'(\beta )\gamma t}\right) (1+ic'(\beta )\gamma t)d\beta . \end{aligned}$$

It has the similar structure as (7.6) and (7.8). The only difference between the first two terms in (7.6) and (8.2) is that $\partial _{\alpha } w(\alpha ,\gamma )$ takes the place of $\frac{z(\alpha ,\gamma )-z(\alpha ,\gamma ')}{\gamma -\gamma '}.$ In (7.8), and the third term of (7.6), $w(\alpha ,\gamma )$ takes the place of $\frac{z(\alpha ,\gamma )-z(\alpha ,\gamma ')}{\gamma -\gamma '}$ and $\int _{0}^{1}\nabla _1 K(\tau (z(\alpha ,\gamma )-z(\beta ,\gamma ))+(1-\tau )(z(\alpha ,\gamma ')-z(\beta ,\gamma ')),\tilde{f}(\alpha )-\tilde{f}(\beta ))d\tau$ is replaced by $\nabla _1 K(z(\alpha ,\gamma )-z(\beta ,\gamma ),\tilde{f}(\alpha )-\tilde{f}(\beta ))$. Therefore we could use the similar estimate and have

$$\begin{aligned} \frac{d}{dt}\Vert w\Vert ^2_{H^3(\mathbb {T})}\lesssim C(\Vert w\Vert ^2_{H^3(\mathbb {T})}). \end{aligned}$$

As in the existence of $z(\alpha ,\gamma ,t)$, we could do the similar energy estimate to the approximation of the equation

8.3 $$\begin{aligned} \frac{dw^n(\alpha ,\gamma ,t)}{dt}=\tilde{T}(w)=\varphi _{n}*(D_zT(z(\alpha ,\gamma ,t),\gamma ,t)[\varphi _n*w^n])+\varphi _{n}*(\partial _{\gamma }T(\alpha ,\gamma ,t)), \end{aligned}$$

with initial value $w^n(\alpha ,\gamma ,0)=0$. Then from the Picard theorem and compactness argument, there exists $0\le t_3\le t_1$, such that

$$\begin{aligned} w(\alpha ,\gamma ,t)=\int _{0}^{t}\tilde{T}(w(\alpha ,\gamma ,\tau ))d\tau , \end{aligned}$$

and

8.4 $$\begin{aligned} \Vert w(\alpha ,\gamma ,t)\Vert _{L^{\infty }([0,t_3],H^3_{\alpha }(\mathbb {T}))}\lesssim 1. \end{aligned}$$

We claim there is an uniform $t_3$ holds for all $\gamma \in [-1,1]$. Moreover, we have the following lemma:

### Lemma 8.1

For any $g(\alpha ),h(\alpha )\in H^{j+1}(\mathbb {T})$, $j\le 2$, we have

8.5 $$\begin{aligned}&\Vert \tilde{T}(g(\alpha ),\gamma ,t)\Vert _{H^j(\mathbb {T})}\lesssim 1, \end{aligned}$$

8.6 $$\begin{aligned}&\Vert \tilde{T}(g(\alpha ),\gamma ,t)-\tilde{T}(h(\alpha ),\gamma ,t)\Vert _{H^j(\mathbb {T})}\lesssim \Vert g(\alpha )-h(\alpha )\Vert _{H^{j+1}(\mathbb {T})}, \end{aligned}$$

8.7 $$\begin{aligned}&\lim _{t\rightarrow t'} \Vert \tilde{T}(g(\alpha ),\gamma ,t)-\tilde{T}(g(\alpha ),\gamma ,t')\Vert _{H^j(\mathbb {T})}=0 . \end{aligned}$$

### Proof

It is easy to get these bounds since $z(\alpha ,\gamma ,t)\in L^{\infty }([0,t_0], H_{\alpha }^5(\mathbb {T}))\cap C^{0}([0,t_0], H_{\alpha }^4(\mathbb {T})).$ $\square$

Then we have $w(\alpha ,\gamma ,t)\in L^{\infty }([0,t_3], H_{\alpha }^3(\mathbb {T}))\cap C^{0}([0,t_3], H_{\alpha }^2(\mathbb {T}))\cap C^{1}([0,t_3], H_{\alpha }^1(\mathbb {T})).$

We claim that we could do the similar argument as in the estimate of $\Vert z(\alpha ,\gamma )-z(\alpha ,\gamma ')\Vert _{H^3_{\alpha }(\mathbb {T})}\lesssim |\gamma -\gamma '|$ to get

$$\begin{aligned} \Vert w(\alpha ,\gamma )-w(\alpha ,\gamma )\Vert _{H^1_{\alpha }(\mathbb {T})}\lesssim |\gamma -\gamma '|. \end{aligned}$$

Then from (8.4), we have

8.8 $$\begin{aligned} \lim _{\gamma '\rightarrow \gamma }\Vert w(\alpha ,\gamma ')-w(\alpha ,\gamma )\Vert _{H^2_{\alpha }(\mathbb {T})}=0. \end{aligned}$$

Now we show *w* is the derivative of *z* with respect of $\gamma$. Let

$$\begin{aligned} v(\alpha ,\gamma ,\gamma ',t)=\frac{z(\alpha ,\gamma ,t)-z(\alpha ,\gamma ',t)}{\gamma -\gamma '}-w(\alpha ,\gamma ,t). \end{aligned}$$

From (8.1) and (7.1), we have

$$\begin{aligned}&\frac{dv(\alpha ,\gamma ,\gamma ',t)}{dt}=\frac{T(z(\alpha ,\gamma ,t),\gamma ,t)-T(z(\alpha ,\gamma ',t),\gamma ,t)}{\gamma -\gamma '}+\frac{\int _{\gamma }^{\gamma '}(\partial _{\eta }T)(z(\alpha ,\gamma ',t),\eta ,t)d\eta }{\gamma -\gamma '}\\&\qquad -D_zT(z(\alpha ,\gamma ,t),\gamma ,t)[w(\alpha ,\gamma ,t)]-(\partial _{\gamma }T)(z(\alpha ,\gamma ,t),\gamma ,t)\\&\quad =\left( \frac{T(z(\alpha ,\gamma ,t),\gamma ,t)-T(z(\alpha ,\gamma ',t),\gamma ,t)}{\gamma -\gamma '}-D_zT(z(\alpha ,\gamma ,t),\gamma ,t)[w(\alpha ,\gamma ,t)]\right) \\&\qquad +\left( \frac{\int _{\gamma }^{\gamma '}(\partial _{\eta }T)(z(\alpha ,\gamma ',t),\eta ,t)d\eta }{\gamma -\gamma '}-(\partial _{\gamma }T)(z(\alpha ,\gamma ,t),\gamma ,t)\right) \\&\quad =Term_1+Term_2. \end{aligned}$$

We have

$$\begin{aligned} Term_2&=\frac{1}{\gamma -\gamma '}\int _{\gamma }^{\gamma '}(\partial _{\eta }T)(z(\alpha ,\gamma ',t),\eta ,t)d\eta -(\partial _{\gamma }T)(z(\alpha ,\gamma ,t),\gamma ,t)\\&=\int _{\gamma }^{\gamma '}\frac{(\partial _{\eta }T)(z(\alpha ,\gamma ',t),\eta ,t)-(\partial _{\gamma }T) (z(\alpha ,\gamma ',t),\gamma ,t)}{\gamma -\gamma '}d\eta \\&\qquad +((\partial _{\gamma }T)(z(\alpha ,\gamma ',t),\gamma ,t) -(\partial _{\gamma }T)(z(\alpha ,\gamma ,t),\gamma ,t)). \end{aligned}$$

From (7.2) and (7.15), we have

$$\begin{aligned}{} & {} \Vert ((\partial _{\gamma }T)(z(\alpha ,\gamma ',t),\gamma ,t)-(\partial _{\gamma }T)(z(\alpha ,\gamma ,t),\gamma ,t))\Vert _{L^2(\mathbb {T})}\\{} & {} \quad \lesssim \Vert z(\alpha ,\gamma ,t)-z(\alpha ,\gamma ',t)\Vert _{H^2(\mathbb {T})}\lesssim |\gamma -\gamma '|, \end{aligned}$$

and

$$\begin{aligned} \Vert (\partial _{\eta }T)(z(\alpha ,\gamma ',t),\eta ,t)-(\partial _{\gamma }T)(z(\alpha ,\gamma ',t),\gamma ,t)\Vert _{L^2(\mathbb {T})}\lesssim |\eta -\gamma |, \end{aligned}$$

Then

$$\begin{aligned} \Vert Term_{2}\Vert _{L^2(\mathbb {T})}\lesssim |\gamma -\gamma '|. \end{aligned}$$

Moreover, for $Term_1$, from (8.2), (7.1), (7.6) and (7.8), we have

$$\begin{aligned}&\frac{T(z(\alpha ,\gamma ,t),\gamma ,t)-T(z(\alpha ,\gamma ',t),\gamma ,t)}{\gamma -\gamma '}-D_zT(z(\alpha ,\gamma ,t),\gamma ,t)[w(\alpha ,\gamma ,t)]\\&\quad =\frac{ic(\alpha )\gamma }{1+ic'(\alpha )\gamma t}\partial _{\alpha }(v(\alpha ,\gamma ,\gamma '))\\&\qquad +\lambda (\alpha )\int _{-\pi }^{\pi }K(z(\alpha ,\gamma )-z(\beta ,\gamma ),\tilde{f}(\alpha ) -\tilde{f}(\beta ))\left( \frac{\partial _{\alpha }v(\alpha ,\gamma ,\gamma ')}{1+ic'(\alpha )\gamma t} -\frac{\partial _{\beta }v(\beta ,\gamma ,\gamma ')}{1+ic'(\beta )\gamma t}\right) \\&\quad (1+ic'(\beta )\gamma t)d\beta \\&\qquad + \lambda (\alpha )\int _{-\pi }^{\pi }\int _{0}^{1}\nabla _1 K(\tau (z(\alpha ,\gamma ) -z(\beta ,\gamma ))\\&\quad +(1-\tau )(z(\alpha ,\gamma ')-z(\beta ,\gamma ')),\tilde{f}(\alpha )-\tilde{f}(\beta ))\nonumber \\&\quad \cdot \left( \frac{z(\alpha ,\gamma )-z(\beta ,\gamma )-(z(\alpha ,\gamma ')-z(\beta ,\gamma '))}{\gamma -\gamma '}\right) \nonumber \\&\qquad \left( \frac{\partial _{\alpha }z(\alpha ,\gamma ')}{1+ic'(\alpha )\gamma t} -\frac{\partial _{\beta }z(\beta ,\gamma ')}{1+ic'(\beta )\gamma t}+\partial _{\alpha }\tilde{f}(\alpha )-\partial _{\beta }\tilde{f}(\beta )\right) (1+ic'(\beta )\gamma t)d\tau d\beta \\&\qquad -\lambda (\alpha )\int _{-\pi }^{\pi } \nabla _1 K(z(\alpha ,\gamma )-z(\beta ,\gamma ),\tilde{f}(\alpha )-\tilde{f}(\beta ))\cdot (w(\alpha ,\gamma )-w(\beta ,\gamma ))\nonumber \\&\quad \cdot \left( \partial _{\alpha }\tilde{f}(\alpha )-\partial _{\beta }\tilde{f}(\beta )+\frac{\partial _{\alpha }z(\alpha ,\gamma )}{1+ic'(\alpha )\gamma t}-\frac{\partial _{\beta }z(\beta ,\gamma )}{1+ic'(\beta )\gamma t}\right) (1+ic'(\beta )\gamma t)d\beta \\&\quad =Term_{1,1}+Term_{1,2}+Term_{1,3}+Term_{1,4}. \end{aligned}$$

Here

$$\begin{aligned}&Term_{1,3}+Term_{1,4}=\lambda (\alpha )\int _{-\pi }^{\pi } \int _{0}^{1}\nabla _1 K(\tau (z(\alpha ,\gamma )-z(\beta ,\gamma ))+(1-\tau )(z(\alpha ,\gamma ')-z(\beta ,\gamma ')),\tilde{f}(\alpha )\\&\quad -\tilde{f}(\beta ))d\tau \cdot (v(\alpha ,\gamma ,\gamma ')-v(\beta ,\gamma ,\gamma '))\nonumber \\&\quad \cdot \left( \partial _{\alpha }\tilde{f}(\alpha )-\partial _{\beta }\tilde{f}(\beta )+\frac{\partial _{\alpha }z(\alpha ,\gamma ')}{1+ic'(\alpha )\gamma t}-\frac{\partial _{\beta }z(\beta ,\gamma ')}{1+ic'(\beta )\gamma t}\right) (1+ic'(\beta )\gamma t) d\beta \\&\quad +\lambda (\alpha )\int _{-\pi }^{\pi }[\int _{0}^{1}\nabla _1 K(\tau (z(\alpha ,\gamma )-z(\beta ,\gamma ))+(1-\tau )(z(\alpha ,\gamma ')-z(\beta ,\gamma ')),\tilde{f}(\alpha )-\tilde{f}(\beta ))d\tau \\&\quad -\nabla _1 K(z(\alpha ,\gamma )-z(\beta ,\gamma ),\tilde{f}(\alpha )-\tilde{f}(\beta ))]\cdot (w(\alpha ,\gamma )-w(\beta ,\gamma ))\nonumber \\&\quad \left( \frac{\partial _{\alpha }z(\alpha ,\gamma ')}{1+ic'(\alpha )\gamma t}-\frac{\partial _{\beta }z(\beta ,\gamma ')}{1+ic'(\beta )\gamma t}+\partial _{\alpha }\tilde{f}(\alpha )-\partial _{\beta }\tilde{f}(\beta )\right) (1+ic'(\beta )\gamma t) d\beta \\&\quad +\lambda (\alpha )\int _{-\pi }^{\pi }\nabla _1 K(z(\alpha ,\gamma )-z(\beta ,\gamma ),\tilde{f}(\alpha )-\tilde{f}(\beta ))\cdot (w(\alpha ,\gamma )-w(\beta ,\gamma ))\nonumber \\&\quad \left( \frac{\partial _{\alpha }z(\alpha ,\gamma ')}{1+ic'(\alpha )\gamma t}-\frac{\partial _{\beta }z(\beta ,\gamma ')}{1+ic'(\beta )\gamma t}-(\frac{\partial _{\alpha }z(\alpha ,\gamma )}{1+ic'(\alpha )\gamma t}-\frac{\partial _{\beta }z(\beta ,\gamma )}{1+ic'(\beta )\gamma t})\right) (1+ic'(\beta )\gamma t) d\beta \\&\quad =Term_{1,5}+Term_{1,6}+Term_{1,7}. \end{aligned}$$

Since the component of $\nabla _1 K$ is of $-2$ type, we could use Lemma 11.2 to bound $Term_{1,5}$ and have

$$\begin{aligned} \Vert Term_{1,5}\Vert _{L^2(\mathbb {T})}^2\lesssim \Vert v(\alpha ,\gamma ,\gamma ')\Vert _{L^2(\mathbb {T})}^2. \end{aligned}$$

For $Term_{1,6}$, we have

$$\begin{aligned}&\Vert \left[ \int _{0}^{1}\nabla _1 K(\tau (z(\alpha ,\gamma )-z(\beta ,\gamma ))+(1-\tau )(z(\alpha ,\gamma ') -z(\beta ,\gamma ')),\tilde{f}(\alpha )-\tilde{f}(\beta ))d\tau \right. \\&\qquad \left. -\nabla _1 K(z(\alpha ,\gamma )-z(\beta ,\gamma ),\tilde{f}(\alpha ) -\tilde{f}(\beta ))\right] (\alpha -\beta )^2\Vert _{C^1([-2\delta ,\delta ]\times [-\pi ,\pi ])}\\&\quad \lesssim \Vert z(\alpha ,\gamma )-z(\alpha ,\gamma ')\Vert _{H^3(\mathbb {T})}. \end{aligned}$$

Then from Lemma 11.3, and (7.15), we have

$$\begin{aligned} \Vert Term_{1,6}\Vert _{L^2(\mathbb {T})}\lesssim |\gamma -\gamma '|. \end{aligned}$$

From Lemma 11.3, we again have

$$\begin{aligned}&\Vert Term_{1,7}\Vert _{L^2(\mathbb {T})}\\&\quad \lesssim \Vert \nabla _1 K(z(\alpha ,\gamma )-z(\beta ,\gamma ),\tilde{f}(\alpha ) -\tilde{f}(\beta ))(\alpha -\beta )^2\Vert _{C^1([-2\delta ,\delta ]\times [-\pi ,\pi ])}\Vert w(\alpha ,\gamma )\Vert _{C^2(\mathbb {T})}\\&\quad \left\| \frac{\partial _{\alpha }z(\alpha ,\gamma ')}{1+ic'(\alpha )\gamma t} -\frac{\partial _{\alpha }z(\alpha ,\gamma )}{1+ic'(\alpha )\gamma t}\right\| _{L^2(\mathbb {T})}\\&\quad \lesssim \Vert z(\alpha ,\gamma )-z(\alpha ,\gamma ')\Vert _{H^1(\mathbb {T})}\lesssim |\gamma -\gamma '|, \end{aligned}$$

where we use (8.4) and (7.15). Therefore we have

$$\begin{aligned}&\frac{d}{dt}\int _{-\pi }^{\pi }|v(\alpha ,\gamma ,\gamma ')|^2d\alpha \\&\quad =2\Re \int _{-\pi }^{\pi }v(\alpha ,\gamma ,\gamma ')\overline{\frac{ic(\alpha )\gamma }{1+ic'(\alpha ) \gamma t}\partial _{\alpha }(v(\alpha ,\gamma ,\gamma '))}d\alpha \\&\qquad +2\Re \int _{-\pi }^{\pi }v(\alpha ,\gamma ,\gamma ')\overline{\lambda (\alpha ) \int _{-\pi }^{\pi }K(z(\alpha ,\gamma )-z(\beta ,\gamma ),\tilde{f}(\alpha ) -\tilde{f}(\beta ))\left( \frac{\partial _{\alpha }v(\alpha ,\gamma ,\gamma ')}{1+ic'(\alpha )\gamma t}-\frac{\partial _{\beta }v(\beta ,\gamma ,\gamma ')}{1+ic'(\beta )\gamma t}\right) (1+ic'(\beta )\gamma t)d\beta }d\alpha \\&\qquad +B.T^0, \end{aligned}$$

where

$$\begin{aligned} B.T^0\lesssim |\gamma -\gamma '|^2+\Vert v(\alpha ,\gamma ,\gamma ')\Vert _{L^2(\mathbb {T})}^2. \end{aligned}$$

Then by corollary 5.6, and the initial value $v(\alpha ,\gamma ,\gamma ',0)=0$, from the Gronwall’s inequality we have $\lim _{\gamma '\rightarrow \gamma }\Vert v(\alpha ,\gamma ,\gamma ',t)\Vert _{L^2(\mathbb {T})}=0$ when $t\le t_3$.

Form (8.4), and (7.15), we have

$$\begin{aligned} \Vert v(\alpha ,\gamma ,\gamma ',t)\Vert _{H^3(\mathbb {T})}\lesssim 1. \end{aligned}$$

Moreover from the interpolation theorem, we have

$$\begin{aligned} \lim _{\gamma '\rightarrow \gamma }\Vert v(\alpha ,\gamma ,\gamma ',t)\Vert _{H^2(\mathbb {T})}=0. \end{aligned}$$

Then from (8.8), we have

$$\begin{aligned} z(\alpha ,\gamma )\in C^1_\gamma ([-1,1],H^2_{\alpha }(\mathbb {T})), \end{aligned}$$

with

$$\begin{aligned} \frac{d}{d\gamma }z(\alpha ,\gamma , t) =w(\alpha ,\gamma ). \end{aligned}$$

From (8.1), we also have

$$\begin{aligned} \frac{d}{dt}\frac{d}{d\gamma }z=\frac{dw}{dt}=\frac{dT(z)}{d\gamma }=\frac{d}{d\gamma }\frac{d}{dt}z. \end{aligned}$$

In conclusion we have

8.9 $$\begin{aligned} {\left\{ \begin{array}{ll} &{}z(\alpha ,\gamma ,t)\in C^1_{t}([0,t_1], H_{\alpha }^3(\mathbb {T})),\\ &{}z(\alpha ,\gamma ,t)\in C^1_\gamma ([-1,1],H^2_{\alpha }(\mathbb {T})),\\ &{}\frac{dz}{d\gamma }\in C^{0}_t([0,t_3], H_{\alpha }^2(\mathbb {T}))\cap C^{1}_t([0,t_3], H_{\alpha }^1(\mathbb {T})),\\ &{}\frac{d}{dt}\frac{d}{d\gamma }z=\frac{d}{d\gamma }\frac{d}{dt}z,\\ &{}z(\alpha ,0,t)=f^c(\alpha ,t), \text { when }0\le t\le t_2. \end{array}\right. } \end{aligned}$$

## The Analyticity

In this section, we want to show $f(\alpha ,t)$ is a real analytic function near 0 for each fixed t, $0< t< t_3$. We first show that it is enough to prove that

9.1 $$\begin{aligned} \frac{ic(\alpha )t}{1+ic^{'}(\alpha )\gamma t}\frac{d}{d\alpha }z(\alpha ,\gamma , t)-\frac{d}{d\gamma }z(\alpha ,\gamma ,t)=0. \end{aligned}$$

### Lemma 9.1

If *z* satisfies (9.1), then *f*(*x*) can be analytically extended to $D_A=\{\alpha +iy|-\infty<\alpha <\infty , -c(\alpha )t\le y\le c(\alpha )t\}.$

### Proof

From the uniqueness (6.5), we have $z(\alpha ,0,t)=f^c(\alpha ,t)$. Then

$$\begin{aligned} f^c(\alpha +iy,t)={\left\{ \begin{array}{ll}&{}z\left( \alpha ,\frac{y}{c(\alpha )t},t\right) ,\ c(\alpha )\ne 0,\\ &{}z(\alpha ,0,t),\ c(\alpha )=0, \end{array}\right. } \end{aligned}$$

is a extension of $f^c(\alpha ,t)$ on $D_A.$ Moreover, when $c(\alpha )\ne 0$, we have

$$\begin{aligned}{} & {} \frac{d}{d\alpha }\left( z\left( \alpha ,\frac{y}{c(\alpha )t},t\right) \right) =(\partial _{\alpha }z)\left( \alpha ,\frac{y}{c(\alpha )t},t\right) -(\partial _{\gamma }z) \left( \alpha ,\frac{y}{c(\alpha )t},t\right) \left( \frac{yc'(\alpha )}{c(\alpha )^2 t}\right) ,\\{} & {} \frac{d}{dy}\left( z\left( \alpha ,\frac{y}{c(\alpha )t},t\right) \right) =(\partial _{\gamma }z)\left( \alpha ,\frac{y}{c(\alpha )t},t\right) \left( \frac{1}{c(\alpha )t}\right) . \end{aligned}$$

Then we have

$$\begin{aligned}&\frac{d}{d\alpha }f^c(\alpha +iy,t)+i\frac{d}{dy}f^c(\alpha +iy,t)\\&\quad =(\partial _{\alpha }z)\left( \alpha ,\frac{y}{c(\alpha )t},t\right) -(\partial _{\gamma }z)\left( \alpha ,\frac{y}{c(\alpha )t},t\right) \left( \frac{yc'(\alpha )}{c(\alpha )^2 t}\right) \\&\quad +i(\partial _{\gamma }z)\left( \alpha ,\frac{y}{c(\alpha )t},t\right) \left( \frac{1}{c(\alpha )t}\right) . \end{aligned}$$

Now let $\frac{y}{c(\alpha )t}=\gamma$. Then

$$\begin{aligned}&\frac{d}{d\alpha }f^c(\alpha +iy,t)+i\frac{d}{dy}f^c(\alpha +iy,t) =\partial _{\alpha }z(\alpha ,\gamma ,t)-\left( \frac{c'(\alpha )\gamma }{c(\alpha )} -\frac{i}{c(\alpha )t}\right) \partial _{\gamma }z(\alpha ,\gamma ,t)\\&\quad =\partial _{\alpha }z(\alpha ,\gamma ,t) -\left( \frac{ic'(\alpha )\gamma t +1}{ic(\alpha )t}\right) \partial _{\gamma }z(\alpha ,\gamma ,t)\\&\quad =0. \end{aligned}$$

Moreover, $z(\alpha ,\gamma ,t)\in C^1_{\gamma }([-1,1],H^2_{\alpha }(\mathbb {T}))$. Then $\partial _{\alpha }f^c$, $\partial _{\gamma }f^c$ are continuous. Therefore we have the analyticity of $f^c$ near 0. We also have $f(\alpha ,t)=f^c(\alpha ,t)$ when $|\alpha |\le \delta$. Then we have the result. $\square$

Let

$$\begin{aligned} A_0(h)(\alpha ,\gamma ,t)=\left( \frac{ic(\alpha )t}{1+ic'(\alpha )\gamma t}\partial _{\alpha }-\partial _{\gamma }\right) h(\alpha ,\gamma ,t). \end{aligned}$$

Before we prove $A_0(z)=0$, we introduce some general lemmas.

### Lemma 9.2

If all the derivatives are well-defined and $\partial _{\alpha }\partial _{\gamma }h(\alpha ,\gamma ,t)=\partial _{\gamma }\partial _{\alpha }h(\alpha ,\gamma ,t)$, we have

$$\begin{aligned} \left( \frac{ic(\alpha )t}{1+ic'(\alpha )\gamma t}\partial _{\alpha }-\partial _{\gamma }\right) \frac{\partial _{\alpha }h(\alpha ,\gamma )}{1+ic'(\alpha )\gamma t}=\frac{\partial _{\alpha }}{1+ic'(\alpha )\gamma t}\left( \frac{ic(\alpha )t}{1+ic'(\alpha )\gamma t}\partial _{\alpha }-\partial _{\gamma }\right) h(\alpha ,\gamma ), \end{aligned}$$

### Proof

First, for the right hand side, we have

$$\begin{aligned} (RHS)&=\frac{\frac{ic(\alpha )t}{1+ic'(\alpha )\gamma t}\partial _{\alpha }^2h(\alpha ,\gamma )}{1+ic'(\alpha )\gamma t}+\frac{ic(\alpha )t}{1+ic'(\alpha )\gamma t}\left( \frac{-ic''(\alpha )\gamma t}{(1+ic'(\alpha )\gamma t )^2}\partial _{\alpha }h(\alpha ,\gamma )\right) \\&\quad -\frac{\partial _{\alpha } \partial _{\gamma }h(\alpha ,\gamma )}{1+ic'(\alpha )\gamma t}+\frac{ic'(\alpha )t\partial _{\alpha }h(\alpha ,\gamma )}{(1+ic'(\alpha )\gamma t)^2}. \end{aligned}$$

Also

$$\begin{aligned} (LHS)&=\left[ \frac{ic'(\alpha )t}{(1+ic'(\alpha )\gamma t)^2}-\frac{ic(\alpha )tic''(\alpha )\gamma t}{(1+ic'(\alpha )\gamma t)^3}\right] \partial _{\alpha }h(\alpha ,\gamma )\\&\quad +\frac{ic(\alpha )t}{(1+ic'(\alpha )\gamma t)^2}\partial _{\alpha }^2h(\alpha ,\gamma )-\frac{1}{1+ic'(\alpha )\gamma t}\partial _{\alpha }\partial _{\gamma }h(\alpha ,\gamma ). \end{aligned}$$

From the two equalities above, we have the result. $\square$

### Lemma 9.3

If all the derivatives are well-defined and we have

$$\begin{aligned} \frac{d}{dt}h= \frac{ic(\alpha )\gamma }{1+ic'(\alpha )\gamma t} \partial _{\alpha }h+\tilde{T}(h), \end{aligned}$$

and $\frac{d}{d\alpha }\frac{d}{dt}h=\frac{d}{dt}\frac{d}{d\alpha }h$, $\frac{d}{dt}\frac{d}{d\gamma }h=\frac{d}{d\gamma }\frac{d}{dt}h$, $\frac{d}{d\alpha }\frac{d}{d\gamma }h=\frac{d}{d\gamma }\frac{d}{d\alpha }h$, then we have

$$\begin{aligned} \frac{d}{dt}A_0(h)= \frac{ic(\alpha )\gamma }{1+ic'(\alpha )\gamma t} \partial _{\alpha }A_0(h)+A_0(\tilde{T}(h)).\end{aligned}$$

### Proof

First,

$$\begin{aligned}&\frac{d}{dt}A_0(h)(\alpha ,\gamma ,t)\\&\quad =\frac{d}{dt}\left( \frac{ic(\alpha )t}{1+ic'(\alpha )\gamma t}\right) \partial _{\alpha }h +\frac{ic(\alpha )t}{1+ic'(\alpha )\gamma t}\partial _{\alpha }\frac{d}{dt}h-\partial _{\gamma }\frac{d}{dt}h\\&\quad =\frac{d}{dt}\left( \frac{ic(\alpha )t}{1+ic'(\alpha )\gamma t}\right) \partial _{\alpha }h +\left( \frac{ic(\alpha )t}{1+ic'(\alpha )\gamma t}\partial _{\alpha }-\partial _{\gamma }\right) \left( \frac{ic(\alpha )\gamma }{1+ic'(\alpha )\gamma t}\partial _{\alpha }h\right) +A_0(\tilde{T}(h))\\&\quad =\frac{d}{dt}\left( \frac{ic(\alpha )t}{1+ic'(\alpha )\gamma t}\right) \partial _{\alpha }h +\frac{ic(\alpha )t}{1+ic'(\alpha )\gamma t}\partial _{\alpha } \left( \frac{ic(\alpha )\gamma }{1+ic'(\alpha )\gamma t}\right) \partial _{\alpha }h +\frac{ic(\alpha )t}{1+ic'(\alpha )\gamma t}\frac{ic(\alpha )\gamma }{1+ic'(\alpha )\gamma t}\partial _{\alpha }^2 h\\&\qquad -\partial _{\gamma }\left( \frac{ic(\alpha )\gamma }{1+ic'(\alpha )\gamma t}\right) \partial _{\alpha }h -(\frac{ic(\alpha )\gamma }{1+ic'(\alpha )\gamma t}\partial _{\gamma }\partial _{\alpha }h)+A_0(\tilde{T}(h))\\&\quad =\left( \underbrace{\frac{ic(\alpha )t}{1+ic'(\alpha )\gamma t}\partial _{\alpha } \left( \frac{ic(\alpha )\gamma }{1+ic'(\alpha )\gamma t}\right) }_{Term_1} +\underbrace{\frac{d}{dt}\left( \frac{ic(\alpha )t}{1+ic'(\alpha )\gamma t}\right) -\partial _{\gamma } \left( \frac{ic(\alpha )\gamma }{1+ic'(\alpha )\gamma t}\right) }_{Term_2}\right) \partial _{\alpha }h\\&\qquad +\underbrace{\frac{ic(\alpha )\gamma }{1+ic'(\alpha )\gamma t} \left( \frac{ic(\alpha )t}{1+ic'(\alpha )\gamma t}\partial _{\alpha }-\partial _{\gamma }\right) \partial _{\alpha }h}_{Term_{3}}+A_0(\tilde{T}(h)). \end{aligned}$$

Moreover, we have

$$\begin{aligned}&\underbrace{\frac{d}{dt}\left( \frac{ic(\alpha )t}{1+ic'(\alpha )\gamma t}\right) -\partial _{\gamma }\left( \frac{ic(\alpha )\gamma }{1+ic'(\alpha )\gamma t}\right) }_{Term_2}=0,\\&\quad \underbrace{\frac{ic(\alpha )t}{1+ic'(\alpha )\gamma t}\partial _{\alpha }\left( \frac{ic(\alpha )\gamma }{1+ic'(\alpha )\gamma t}\right) }_{Term_1}= \frac{ic(\alpha )\gamma }{1+ic'(\alpha )\gamma t}\partial _{\alpha }\left( \frac{ic(\alpha )t}{1+ic'(\alpha )\gamma t}\right) , \end{aligned}$$

and

$$\begin{aligned}&\underbrace{\frac{ic(\alpha )\gamma }{1+ic'(\alpha )\gamma t} \left( \frac{ic(\alpha )t}{1+ic'(\alpha )\gamma t}\partial _{\alpha }-\partial _{\gamma }\right) \partial _{\alpha }h}_{Term_3}\\&\quad = \frac{ic(\alpha )\gamma }{1+ic'(\alpha )\gamma t}\partial _{\alpha } \left( \left( \frac{ic(\alpha )t}{1+ic'(\alpha )\gamma t}\partial _{\alpha }-\partial _{\gamma }\right) h\right) \\&\quad -\frac{ic(\alpha )\gamma }{1+ic'(\alpha )\gamma t}\partial _{\alpha } \left( \frac{ic(\alpha )t}{1+ic'(\alpha )\gamma t}\right) \partial _{\alpha }h. \end{aligned}$$

Therefore

$$\begin{aligned} \frac{d}{dt}A_0(h)(\alpha ,\gamma ,t)= & {} \frac{ic(\alpha )\gamma }{1+ic'(\alpha )\gamma t}\partial _{\alpha }\left( \left( \frac{ic(\alpha )t}{1+ic'(\alpha )\gamma t}\partial _{\alpha }-\partial _{\gamma }\right) h\right) +A_0(\tilde{T}(h))\\= & {} \frac{ic(\alpha )\gamma }{1+ic'(\alpha )\gamma t}\partial _{\alpha }A_0(h)+A_0(\tilde{T}(h)). \end{aligned}$$

$$\square $$

### Lemma 9.4

Let $\tilde{K}$ be meromorphic. $\partial _{\alpha }X(\alpha ,\gamma )$, $\partial _{\gamma }X(\alpha ,\gamma )$ are well-defined and in $C_{\alpha }^{0}[-\pi ,\pi ]$, $\partial _{\alpha }h(\alpha ,\gamma )$ and $\partial _{\gamma }h(\alpha ,\gamma )$ are well-defined vector functions with components in $C_{\alpha }^{0}[-\pi ,\pi ]$. If for fixed $\alpha$, there is no singular point in the integrals below and $c(\pi )=c(-\pi )=0$, $c(\alpha )\in W^{2,\infty }$, then we have

$$\begin{aligned}&A_0(\int _{-\pi }^{\pi }\tilde{K}(h(\alpha ,\gamma )-h(\beta ,\gamma ))X(\beta ,\gamma )(1+ic'(\beta )\gamma t)d\beta )\\&\quad =\int _{-\pi }^{\pi }\nabla \tilde{K}(h(\alpha ,\gamma )-h(\beta ,\gamma ))\cdot (A_0(h)(\alpha ,\gamma )-A_0(h)(\beta ,\gamma ))X(\beta ,\gamma )(1+ic'(\beta )\gamma t)d\beta \\&\qquad +\int _{-\pi }^{\pi }\tilde{K}(h(\alpha ,\gamma )-h(\beta ,\gamma ))A_0(X)(\beta ,\gamma )(1+ic'(\beta )\gamma t)d\beta \\&\quad =D_{h}(\int _{-\pi }^{\pi }\tilde{K}(h(\alpha ,\gamma )-h(\beta ,\gamma ))X(\beta ,\gamma )(1+ic'(\beta )\gamma t)d\beta )[A_0(h)]\\&\qquad +\int _{-\pi }^{\pi }\tilde{K}(h(\alpha ,\gamma )-h(\beta ,\gamma ))A_0(X)(\beta ,\gamma )(1+ic'(\beta )\gamma t)d\beta . \end{aligned}$$

Here $D_h$ is the Gateaux derivative.

### Proof

We have

$$\begin{aligned}&A_0\left( \int _{\alpha -\pi }^{\alpha +\pi }\tilde{K}(h(\alpha ,\gamma )-h(\alpha -\beta ,\gamma ))X(\alpha -\beta ,\gamma )(1+ic'(\alpha -\beta )\gamma t)d\beta \right) \\&\quad =\underbrace{\frac{ic(\alpha )t}{1+ic'(\alpha )\gamma t}(\tilde{K}(h(\alpha ,\gamma )-h(-\pi ,\gamma ))X(-\pi ,\gamma )(1+ic'(-\pi )\gamma t)-\tilde{K}(h(\alpha ,\gamma )-h(\pi ,\gamma ))X(\pi ,\gamma )(1+ic'(\pi )\gamma t))}_{Term_1}\\&\qquad +\underbrace{\int _{\alpha -\pi }^{\alpha +\pi }\nabla \tilde{K}(h(\alpha ,\gamma ) -h(\alpha -\beta ,\gamma ))\cdot \left( \left( \frac{ic(\alpha )t}{1+ic'(\alpha )\gamma t}\partial _{\alpha }-\partial _{\gamma }\right) h(\alpha ,\gamma )-(\frac{ic(\alpha )t}{1+ic'(\alpha )\gamma t}\partial _{\alpha }-\partial _{\gamma })h(\alpha -\beta ,\gamma ,t)\right) }_{Term_2}\\&\qquad \underbrace{X(\alpha -\beta ,\gamma )(1+ic'(\alpha -\beta )\gamma t)d\beta }_{Term_2}\\&\qquad +\underbrace{\int _{\alpha -\pi }^{\alpha +\pi }\tilde{K}(h(\alpha ,\gamma )-h(\alpha -\beta ,\gamma )) \left( \frac{ic(\alpha )t}{1+ic'(\alpha )\gamma t}\partial _{\alpha }-\partial _{\gamma }\right) X(\alpha -\beta ,\gamma ) (1+ic'(\alpha -\beta )\gamma t)d\beta }_{Term_3}\\&\qquad +\underbrace{\int _{\alpha -\pi }^{\alpha +\pi }\tilde{K}(h(\alpha ,\gamma )-h(\alpha -\beta ,\gamma )) X(\alpha -\beta ,\gamma )\left( \frac{ic(\alpha )t}{1+ic'(\alpha )\gamma t}ic'' (\alpha -\beta )\gamma t-ic'(\alpha -\beta ) t\right) d\beta }_{Term_4}\\&\quad =Term 1 +\\&\qquad +\underbrace{\int _{\alpha -\pi }^{\alpha +\pi }\nabla \tilde{K}(h(\alpha ,\gamma ) -h(\alpha -\beta ,\gamma ))\cdot \left( \left( \frac{ic(\alpha )t}{1+ic'(\alpha )\gamma t}\partial _{\alpha } -\partial _{\gamma }\right) h(\alpha ,\gamma )-\left( \frac{ic(\alpha -\beta )t}{1+ic'(\alpha -\beta )\gamma t} \partial _{\alpha }-\partial _{\gamma }\right) h(\alpha -\beta ,\gamma ,t)\right) }_{Term_{2,1}}\\&\qquad \underbrace{X(\alpha -\beta ,\gamma )(1+ic'(\alpha -\beta )\gamma t)d\beta }_{Term_{2,1}}\\&\qquad +\underbrace{\int _{\alpha -\pi }^{\alpha +\pi }\nabla \tilde{K}(h(\alpha ,\gamma ) -h(\alpha -\beta ,\gamma ))\cdot ((\frac{ic(\alpha -\beta )t}{1+ic'(\alpha -\beta )\gamma t}\partial _{\alpha }-\frac{ic(\alpha )t}{1+ic'(\alpha )\gamma t}\partial _{\alpha })h(\alpha -\beta ,\gamma ,t))}_{Term_{2,2}}\\&\qquad \underbrace{X(\alpha -\beta ,\gamma )(1+ic'(\alpha -\beta )\gamma t)d\beta }_{Term_{2,2}}\\&\qquad +\underbrace{\int _{\alpha -\pi }^{\alpha +\pi }\tilde{K}(h(\alpha ,\gamma )-h(\alpha -\beta ,\gamma )) \left( \frac{ic(\alpha -\beta )t}{1+ic'(\alpha -\beta )\gamma t}\partial _{\alpha }-\partial _{\gamma }\right) X(\alpha -\beta ,\gamma )(1+ic'(\alpha -\beta )\gamma t)d\beta }_{Term_{3,1}}\\&\qquad +\underbrace{\int _{\alpha -\pi }^{\alpha +\pi }\tilde{K}(h(\alpha ,\gamma )-h(\alpha -\beta ,\gamma )) \left( \frac{ic(\alpha )t}{1+ic'(\alpha )\gamma t}\partial _{\alpha } -\frac{ic(\alpha -\beta )t}{1+ic'(\alpha -\beta )\gamma t} \partial _{\alpha }\right) X(\alpha -\beta ,\gamma )(1+ic'(\alpha -\beta )\gamma t)d\beta }_{Term_{3,2}}\\&\qquad +\underbrace{\int _{\alpha -\pi }^{\alpha +\pi }\tilde{K}(h(\alpha ,\gamma ) -h(\alpha -\beta ,\gamma ))X(\alpha -\beta ,\gamma ) \left( \frac{ic(\alpha )t}{1+ic'(\alpha )\gamma t}ic''(\alpha -\beta )\gamma t-ic'(\alpha -\beta ) t\right) d\beta }_{Term_4}\\&\quad =Term_1+Term_2+Term_{2,2}+Term_{3,1}+Term_{3,2}+Term_4. \end{aligned}$$

Moreover,

$$\begin{aligned}&Term_{2,2}+Term_{3,2}+Term_4\\&\quad =\int _{\alpha -\pi }^{\alpha +\pi }\frac{d}{d\beta }(\tilde{K}(h(\alpha ,\gamma )-h(\alpha -\beta ,\gamma )) X(\alpha -\beta ,\gamma )(ic(\alpha -\beta )t\\&\qquad -\frac{ic(\alpha )t}{1+ic'(\alpha )\gamma t}(1+ic'(\alpha -\beta )\gamma t)))d\beta \\&\quad =\tilde{K}(h(\alpha ,\gamma )-h(-\pi ,\gamma ))X(-\pi ,\gamma )(ic(-\pi )t-\frac{ic(\alpha )t}{1+ic'(\alpha )\gamma t}(1+ic'(-\pi )\gamma t))\\&\qquad -\tilde{K}(h(\alpha ,\gamma )-h(\pi ,\gamma ))X(\pi ,\gamma )(ic(\pi )t-\frac{ic(\alpha )t}{1+ic'(\alpha )\gamma t}(1+ic'(\pi )\gamma t)). \end{aligned}$$

We use the condition that $c(-\pi )=c(\pi )=0$ and we could get the $Term_1+Term_{2,2}+Term_{3,2}+Term_4=0$. Then we have the result. $\square$

Now we use Lemmas 9.2, 9.3, 9.4, to show the result. From (4.5) and (5.5), we have

$$\begin{aligned}&\frac{d z_{\mu }(\alpha , \gamma ,t)}{dt}=\frac{ic(\alpha )\gamma }{1+ic^{'} (\alpha )\gamma t}\partial _{\alpha }z_{\mu }(\alpha ,\gamma ,t)\\&\quad +\lambda (\alpha _{\gamma }^{t})\int _{-\pi }^{\pi }K(z(\alpha ,\gamma ,t)-z(\beta ,\gamma ,t), \tilde{f}(\alpha _{\gamma }^t,t)-\tilde{f}(\beta _{\gamma }^t,t))\\&\quad \left( \frac{\partial _{\alpha }z_{\mu }(\alpha ,\gamma ,t)}{1+ic'(\alpha )\gamma t} -\frac{\partial _{\beta }z_{\mu }(\beta ,\gamma ,t)}{1+ic'(\beta )\gamma t}\right) (1+ic'(\beta )\gamma t)d\beta \\&\quad +\lambda (\alpha _{\gamma }^t)\int _{-\pi }^{\pi }K(z(\alpha ,\gamma ,t) -z(\beta ,\gamma ,t),\tilde{f}(\alpha _{\gamma }^t,t)-\tilde{f}(\beta _{\gamma }^t,t)) ((\partial _{\alpha }\tilde{f_{\mu }})(\alpha _{\gamma }^t,t)\\&\quad -(\partial _{\beta }\tilde{f_{\mu }})(\beta _{\gamma }^t,t))(1+ic'(\beta )\gamma t)d\beta . \end{aligned}$$

Since we have $A_0(\lambda (\alpha _{\gamma }^{t}))=0$, from Lemma 9.3, we get

$$\begin{aligned}&\frac{dA_0(z_{\mu })}{dt}=\frac{ic(\alpha )\gamma }{1+ic^{'}(\alpha )\gamma t}\partial _{\alpha }A_0(z_{\mu })(\alpha ,\gamma ,t)\\&\quad +\lambda (\alpha _{\gamma }^{t})A_0(\int _{-\pi }^{\pi }K(z(\alpha ,\gamma ,t)-z(\beta ,\gamma ,t),\tilde{f}(\alpha _{\gamma }^t,t)-\tilde{f}(\beta _{\gamma }^t,t))\\&\quad \left( \frac{\partial _{\alpha }z_{\mu }(\alpha ,\gamma ,t)}{1+ic'(\alpha )\gamma t} -\frac{\partial _{\beta }z_{\mu }(\beta ,\gamma ,t)}{1+ic'(\beta )\gamma t}+(\partial _{\alpha }\tilde{f_{\mu }})(\alpha _{\gamma }^t,t)-(\partial _{\beta }\tilde{f_{\mu }})(\beta _{\gamma }^t,t)\right) (1+ic'(\beta )\gamma t)d\beta ). \end{aligned}$$

Let

$$\begin{aligned} h(\alpha ,\gamma )= & {} (z(\alpha ,\gamma ,t),\tilde{f}(\alpha _{\gamma }^t,t), \frac{\partial _{\alpha }z_{\mu }(\alpha ,\gamma ,t)}{1+ic'(\alpha )\gamma t}+(\partial _{\alpha }\tilde{f_{\mu }})(\alpha _{\gamma }^t,t)),\\ \tilde{K}(h(\alpha ,\gamma )-h(\beta ,\gamma ))= & {} K(h_1(\alpha ,\gamma )-h_1(\beta ,\gamma ),h_2(\alpha ,\gamma )\\{} & {} -h_2(\beta ,\gamma ))(h_3(\alpha ,\gamma )-h_3(\beta ,\gamma )), \end{aligned}$$

and

$$\begin{aligned} X(\alpha ,\gamma )=1. \end{aligned}$$

then by Lemmas 9.4 and 9.2 and $A_0((\partial _{\alpha }\tilde{f_{\mu }})(\alpha _{\gamma }^t,t))=0$, and $A_0(\tilde{f_{\mu }}(\alpha _{\gamma }^t,t))=0$, we have

$$\begin{aligned}&\frac{dA_0(z_{\mu })}{dt}=\frac{ic(\alpha )\gamma }{1+ic^{'}(\alpha )\gamma t}\partial _{\alpha }A_0(z_{\mu })(\alpha ,\gamma ,t)\\&\qquad +\lambda (\alpha _{\gamma }^{t})\int _{-\pi }^{\pi }K(z(\alpha ,\gamma ,t)-z(\beta ,\gamma ,t), \tilde{f}(\alpha _{\gamma }^t,t)-\tilde{f}(\beta _{\gamma }^t,t))\\&\qquad \left( \frac{\partial _{\alpha }A_0(z_{\mu })(\alpha ,\gamma ,t)}{1+ic'(\alpha )\gamma t}-\frac{\partial _{\beta }A_0(z_{\mu })(\beta ,\gamma ,t)}{1+ic'(\beta )\gamma t}\right) \\&\quad \cdot (1+ic'(\beta )\gamma t)d\beta \\&\qquad +\lambda (\alpha _{\gamma }^{t})\int _{-\pi }^{\pi }\nabla _1 K(z(\alpha ,\gamma ,t)-z(\beta ,\gamma ,t),\tilde{f}(\alpha _{\gamma }^t,t)-\tilde{f}(\beta _{\gamma }^t,t))\\&\qquad \cdot (A_0(z)(\alpha ,\gamma ,t)-A_0(z)(\beta ,\gamma ,t))\\&\quad \cdot \left( \frac{\partial _{\alpha }z_{\mu }(\alpha ,\gamma ,t)}{1+ic'(\alpha )\gamma t}-\frac{\partial _{\beta }z_{\mu }(\beta ,\gamma ,t)}{1+ic'(\beta )\gamma t}+(\partial _{\alpha }\tilde{f_{\mu }})(\alpha _{\gamma }^t,t)-(\partial _{\beta }\tilde{f_{\mu }})(\beta _{\gamma }^t,t)\right) \\&\quad (1+ic'(\beta )\gamma t)d\beta \\&\quad =Term_1+Term_2+Term_3. \end{aligned}$$

Since

$$\begin{aligned}{} & {} \Vert \nabla _1 K(z(\alpha ,\gamma ,t)-z(\beta ,\gamma ,t)+\tilde{f}(\alpha _{\gamma }^t,t)-\tilde{f}(\beta _{\gamma }^t,t))(\alpha -\beta )^2\Vert _{C^1([-2\delta ,2\delta ]\times [-\pi ,\pi ])}\lesssim 1,\\{} & {} \quad \left\| \frac{\partial _{\alpha }z_{\mu }(\alpha ,\gamma ,t)}{1+ic'(\alpha )\gamma t} +(\partial _{\alpha }\tilde{f_{\mu }})(\alpha _{\gamma }^t,t)\right\| _{C^2([-2\delta ,2\delta ]\times [-\pi ,\pi ])}\lesssim 1, \end{aligned}$$

by Lemma 11.3, we have

$$\begin{aligned} \Vert Term_3\Vert _{L^2(\mathbb {T})}\lesssim \Vert A_0(z)\Vert _{L^2(\mathbb {T})}. \end{aligned}$$

Then by corollary 5.6, we have

$$\begin{aligned} \frac{d}{dt}\Vert A_0(z)\Vert _{L^2(\mathbb {T})}^2\lesssim \Vert A_0(z)\Vert _{L^2(\mathbb {T})}^2. \end{aligned}$$

Moreover when $t=0$, $A_0(z)=-\partial _{\gamma }z(\alpha ,\gamma ,0)=-\partial _{\gamma }f(\alpha ,0)=0$. Therefore $A_0(z)=0$, when $t\le t_3$.

## Using the Energy Estimate to Show the Analyticity

Following an idea similar to that in the previous sections, we introduce a way to study the analyticity of the solution to some differential equations, which is, to our knowledge, a new method.

### Theorem 10.1

Let *T*(*f*) be an operator satisfying the conditions below. We assume that there exists $\epsilon >0$, $k\ge 1$, $f_0\in H^{k}(\mathbb {T})$, such that when $\Vert f-f_0\Vert _{H^{k}}\lesssim \epsilon$,

- (Boundedness) $$T(f):H^{k}(\mathbb {T})\rightarrow H^{k}(\mathbb {T})$$ with $$\Vert T(f)\Vert _{H^{k}(\mathbb {T})}\lesssim 1$$,
- (Existence and boundedness of the Fréchet derivative )$\Vert D_{f}(T(f))[h]\Vert _{H^{k}(\mathbb {T})}\lesssim \Vert h\Vert _{H^k(\mathbb {T})},$
- ($L^2$ boundedness of the Fréchet derivative) $\Vert D_{f}(T(f))[h]\Vert _{L^{2}(\mathbb {T})}\lesssim \Vert h\Vert _{L^2(\mathbb {T})}$,
- $$\frac{d}{dx}T(f)=D_{f}(T(f))[\frac{df}{dx}]$$,
- $$iD_f(T(f))[h]=D_f(T(f))[ih]$$.

Here $\mathbb {T}$ is the torus of length $2\pi$. If $f_0(x)$ also satisfies the equation

10.1 $$\begin{aligned} f_0^{'}(x)=T(f_0), \end{aligned}$$

$f_{0}(x)$ must be real analytic.

### Proof

First, we assume $f_0$ to be an analytic function with analytic continuation *f*(*x*, *t*). Then through the Cauchy-Riemann equation and (10.1), we have

10.2 $$\begin{aligned} {\left\{ \begin{array}{ll} \frac{d}{dt}f(x,t)=iT(f(x,t)),\\ f(x,0)=f_0(x). \end{array}\right. } \end{aligned}$$

Our goal is to show this solution *f*(*x*, *t*) does exist and is analytic.

Through (a), (b), we have

$$\begin{aligned} \Vert T(f)-T(g)\Vert _{H^{k}(\mathbb {T})}\lesssim \Vert f-g\Vert _{H^k(\mathbb {T})}. \end{aligned}$$

We can use the Picard theorem to show there is a solution satisfying

$$\begin{aligned} f(x,t)-f(x,0)=\int _{0}^{t}iT(f(x,\tau ))d\tau . \end{aligned}$$

with $|t|<t_0$ for some $t_0>0$. Moreover

10.3 $$\begin{aligned} f(x,t)\in W^{2,\infty }((-t_0, t_0),H^k(\mathbb {T})). \end{aligned}$$

By (10.3), we have

$$\begin{aligned} \lim _{\Delta t \rightarrow 0}\left\| \frac{f(x,t)-f(x,t+\Delta t)}{\Delta t}-\frac{d}{dt}f(x,t)\right\| _{H^{k}(\mathbb {T})}=0. \end{aligned}$$

Hence

$$\begin{aligned} \lim _{\Delta t \rightarrow 0}\left\| \frac{\frac{d}{dx}f(x,t)-\frac{d}{dx}f(x,t+\Delta t)}{\Delta t}-\frac{d}{dx}\frac{d}{dt}f(x,t)\right\| _{H^{k-1}(\mathbb {T})}=0. \end{aligned}$$

Therefore we have

10.4 $$\begin{aligned} \frac{d}{dt}\frac{d}{dx}f(x,t)=\frac{d}{dx}\frac{d}{dt}f(x,t)\in C^{0}((-t_0, t_0),H^{k-1}(\mathbb {T}))\subset C^{0}((-t_0, t_0),L^2(\mathbb {T})), \end{aligned}$$

and

10.5 $$\begin{aligned} \frac{d}{dx}f(x,t)+i\frac{d}{dt}f(x,t)\in W^{1,\infty }((-t_0,t_0), L^2(\mathbb {T})). \end{aligned}$$

Then we can control $\Vert \frac{d}{dx}f(x,t)+i\frac{d}{dt}f(x,t)\Vert _{L^2(\mathbb {T})}$. We have

10.6 $$\begin{aligned}&\left| \frac{d}{dt}\int _{-\pi }^{\pi }\right| \left( \frac{d}{dx}+i\frac{d}{dt}\right) f(x,t)|^2dx|\nonumber \\&\quad =|2Re\int _{-\pi }^{\pi }\left( \frac{d}{dx}+i\frac{d}{dt}\right) f(x,t)\overline{\frac{d}{dt} \left( \frac{d}{dx}+i\frac{d}{dt}\right) f(x,t)}dx|\nonumber \\&\quad =|2Re\int _{-\pi }^{\pi }\left( \frac{d}{dx}+i\frac{d}{dt}\right) f(x,t) \overline{\left( \frac{d}{dx}+i\frac{d}{dt}\right) \frac{d}{dt}f(x,t)}dx|\nonumber \\&\quad =|2Re\int _{-\pi }^{\pi }\left( \frac{d}{dx}+i\frac{d}{dt}\right) f(x,t) \overline{\left( \frac{d}{dx}+i\frac{d}{dt}\right) iT(f(x,t))}dx|\nonumber \\&\quad =|2Re\int _{-\pi }^{\pi }\left( \frac{d}{dx}+i\frac{d}{dt}\right) f(x,t) \overline{iD_{f}T(f(x,t))[\left( \frac{d}{dx}+i\frac{d}{dt}\right) [f(x,t)]}dx|\nonumber \\&\quad \lesssim \int _{-\pi }^{\pi }\left| \left( \frac{d}{dx}+i\frac{d}{dt}\right) f(x,t)\right| ^2dx. \end{aligned}$$

Here the first equality follows from (10.5), the second from (10.4).

Through (10.1), (10.2), we have

$$\begin{aligned} \left\| \frac{d}{dx}f(x,t)+i\frac{d}{dt}f(x,t)\right\| _{L^2(\mathbb {T})}|_{t=0}=0. \end{aligned}$$

Moreover, from(10.5), $\Vert \frac{d}{dx}f(x,t)+i\frac{d}{dt}f(x,t)\Vert _{L^2(\mathbb {T})}^2\in W^{1,\infty }(-t_0,t_0).$ Then

$$\begin{aligned} \left\| \frac{d}{dx}f(x,t)+i\frac{d}{dt}f(x,t)\right\| _{L^2(\mathbb {T})}^2 =\int _{0}^{t}\frac{d}{d\tau }\left\| \frac{d}{dx}f(x,\tau )+i\frac{d}{d\tau }f(x,\tau )\right\| _{L^2(\mathbb {T})}^2d\tau . \end{aligned}$$

Hence we can use the Gronwall inequality and get

10.7 $$\begin{aligned} \left\| \frac{d}{dx}f(x,t)+i\frac{d}{dt}f(x,t)\right\| _{L^2(\mathbb {T})}=0. \end{aligned}$$

Moreover, since $k\ge 1$, from (10.3), we have $\frac{d}{dt}f(x,t)\in W^{1,\infty }((-t_0,t_0),H^{1}(\mathbb {T}))$. Then $\frac{d}{dt}f(x,t)$ is continuous in *x* and *t*. Therefore $\partial _{x}f(x,t)$ is continuous in *x* and *t*.

Then by the (10.7) and (10.2), we have the analyticity. $\square$

## Data Availability

Data sharing not applicable to this article as no datasets were generated or analysed during the current study.

## Appendix

### Lemma 11.1

For $G(\alpha ,\beta )\in C^1([-2\delta ,2\delta ]\times [-\pi ,\pi ])$, we have

$$\begin{aligned}&\Vert p.v.\int _{-\pi }^{\pi }\frac{G(\alpha ,\beta )}{(\alpha -\beta )}d\beta \Vert _{L^\infty [-2\delta ,2\delta ]}\lesssim \Vert G(\alpha ,\beta )\Vert _{C^1([-2\delta ,2\delta ]\times [-\pi ,\pi ])}. \end{aligned}$$

### Proof

We have

$$\begin{aligned}&\left| p.v.\int _{-\pi }^{\pi }\frac{G(\alpha ,\beta )}{(\alpha -\beta )}d\beta \right| \\&\quad =\left| \int _{-\pi }^{\pi }\frac{G(\alpha ,\beta )-G(\alpha ,\alpha )}{(\alpha -\beta )}d\beta \right| \\&\qquad +\left| p.v.\int _{-\pi }^{\pi }\frac{1}{\alpha -\beta }d\beta G(\alpha ,\alpha )\right| \lesssim \Vert G(\alpha ,\beta )\Vert _{C^1([-2\delta ,2\delta ]\times [-\pi ,\pi ])}. \end{aligned}$$

$$\square $$

### Lemma 11.2

For $g(\alpha )\in L^2[-\pi ,\pi ]$, $G(\alpha ,\beta )\in C^1([-2\delta ,2\delta ]\times [-\pi ,\pi ])$, we have

$$\begin{aligned}&\left\| \int _{-\pi }^{\pi }G(\alpha ,\beta )\frac{g(\alpha )-g(\beta )}{(\alpha -\beta )}d\beta \right\| _{L^2[-2\delta ,2\delta ]}\lesssim \Vert G(\alpha ,\beta )\Vert _{C^1([-2\delta ,2\delta ]\times [-\pi ,\pi ])}\Vert g\Vert _{L^2[-\pi ,\pi ]}. \end{aligned}$$

### Proof

We have

$$\begin{aligned}&\int _{-\pi }^{\pi }G(\alpha ,\beta )\frac{g(\alpha )-g(\beta )}{(\alpha -\beta )}d\beta \\&\quad =\int _{-\pi }^{\pi }\frac{G(\alpha ,\beta )-G(\alpha ,\alpha )}{\alpha -\beta }(g(\alpha )-g(\beta ))d\beta +G(\alpha ,\alpha )p.v.\int _{-\pi }^{\pi }\frac{g(\alpha )-g(\beta )}{\alpha -\beta }d\beta \\&\quad =Term_{1}+Term_{2}. \end{aligned}$$

Here

$$\begin{aligned} \Vert Term_1\Vert _{L^2[-2\delta ,2\delta ]}\lesssim \Vert G(\alpha ,\beta )\Vert _{C^1([-2\delta ,2\delta ]\times [-\pi ,\pi ])}\Vert g(\alpha )\Vert _{L^2[-\pi ,\pi ]}, \end{aligned}$$

and

$$\begin{aligned} \Vert Term_2\Vert _{L^2[-2\delta ,2\delta ]}\lesssim \Vert G(\alpha ,\alpha )\Vert _{C^0([-2\delta ,2\delta ])}\Vert g(\alpha )\Vert _{L^2[-\pi ,\pi ]}. \end{aligned}$$

$$\square $$

### Corollary 11.3

For $g(\alpha )\in L^2[-\pi ,\pi ]$, $h(\alpha )\in C^2[-\pi ,\pi ]$, $G(\alpha ,\beta )\in C^1([-2\delta ,2\delta ]\times [-\pi ,\pi ])$, we have

$$\begin{aligned}&\left\| \int _{-\pi }^{\pi }G(\alpha ,\beta )\frac{(g(\alpha )-g(\beta )) (h(\alpha )-h(\beta ))}{(\alpha -\beta )^2}d\beta \right\| _{L^2[-2\delta ,2\delta ]}\\&\quad \lesssim \Vert G(\alpha ,\beta )\Vert _{C^1([-2\delta ,2\delta ]\times [-\pi ,\pi ])}\Vert g\Vert _{L^2[-2\delta ,2\delta ]}\Vert h\Vert _{C^2[-\pi ,\pi ]}. \end{aligned}$$

### Proof

We can use Lemma 11.2 and let $\tilde{G}(\alpha ,\beta )=G(\alpha ,\beta )\frac{h(\alpha )-h(\beta )}{(\alpha -\beta )}.$ $\square$

### Lemma 11.4

For $g(\alpha )\in H^1[-\pi ,\pi ]$, $G(\alpha ,\beta )\in C^0([-2\delta ,2\delta ]\times [-\pi ,\pi ])$, we have

$$\begin{aligned}&\left\| \int _{-\pi }^{\pi }G(\alpha ,\beta )\frac{g(\alpha )-g(\beta )}{(\alpha -\beta )}d\beta \right\| _{L^2[-2\delta ,2\delta ]}\lesssim \Vert G(\alpha ,\beta )\Vert _{C^0([-2\delta ,2\delta ]\times [-\pi ,\pi ])}\Vert g\Vert _{H^1[-\pi ,\pi ]}. \end{aligned}$$

### Proof

We have

$$\begin{aligned}&\left| \int _{-\pi }^{\pi }G(\alpha ,\beta )\frac{g(\alpha )-g(\beta )}{(\alpha -\beta )}d\beta \right| \\&\quad \le \Vert G(\alpha ,\beta )\Vert _{C^0([-2\delta ,2\delta ]\times [-\pi ,\pi ])}\int _{-\pi }^{\pi }\frac{|g(\alpha )-g(\beta )|}{(\alpha -\beta )}d\beta \\&\quad \le \Vert G(\alpha ,\beta )\Vert _{C^0([-2\delta ,2\delta ]\times [-\pi ,\pi ])}\int _{-\pi }^{\pi }\int _{0}^1|g'(\tau (\alpha )+(1-\tau )(\beta ))|d\tau d\beta \\&\quad \lesssim \Vert G(\alpha ,\beta )\Vert _{C^0([-2\delta ,2\delta ]\times [-\pi ,\pi ])}\Vert g\Vert _{H^1[-\pi ,\pi ]}. \end{aligned}$$

$$\square $$

### Lemma 11.5

For $g(\alpha )\in H^k(\mathbb {T})$, $G(\alpha ,\beta )\in C^1([-2\delta ,2\delta ]\times \mathbb {T})\cap C^k([-2\delta ,2\delta ]\times \mathbb {T})$, for $k\ge 0$, we have

$$\begin{aligned}&\left\| \int _{-\pi }^{\pi }G(\alpha ,\beta )\frac{g(\alpha )-g(\beta )}{\alpha -\beta }d\beta \right\| _{H^{k}[-2\delta ,2\delta ]}\\&\quad \lesssim (\Vert G(\alpha ,\beta )\Vert _{C^1([-2\delta ,2\delta ]\times \mathbb {T})}+\Vert G(\alpha ,\beta )\Vert _{C^k([-2\delta ,2\delta ]\times \mathbb {T})})\Vert g\Vert _{H^k(\mathbb {T})}. \end{aligned}$$

### Proof

From Lemma 11.2, when $k=0$, we have the $L^2$ norm. Moreover, when $k\ge 1$, we have

$$\begin{aligned}&\partial _{\alpha }^{k}\int _{-\pi }^{\pi }G(\alpha ,\beta )\frac{g(\alpha )-g(\beta )}{\alpha -\beta }d\beta \\&\quad =\partial _{\alpha }^{k}\int _{-\pi }^{\pi }G(\alpha ,\alpha -\beta )\frac{g(\alpha )-g(\alpha -\beta )}{\beta }d\beta \\&\quad =\sum _{0\le j \le k} C_j \int _{-\pi }^{\pi }\partial _{\alpha }^j G(\alpha ,\alpha -\beta )\frac{\partial _{\alpha }^{k-j}g(\alpha )-\partial _{\alpha }^{k-j}g(\alpha -\beta )}{\beta }d\beta . \end{aligned}$$

For $j\le k-1$, we could use Lemma 11.2 to get the estimate. For $j= k$, we could use Lemma 11.4 to get the estimate. $\square$

### Lemma 11.6

For $k\ge 2$, $g(\alpha )\in H^{k}(\mathbb {T})$, $G(\alpha ,\beta )\in C^k([-2\delta ,2\delta ]\times \mathbb {T})$, we have

$$\begin{aligned}&\left\| \partial _{\alpha }^{k}\left( \int _{-\pi }^{\pi }G(\alpha ,\beta )\frac{g(\alpha )-g(\beta )}{\alpha -\beta }d\beta \right) -\int _{-\pi }^{\pi }G(\alpha ,\beta )\frac{\partial _{\alpha }^{k}g(\alpha ) -\partial _{\beta }^{k}g(\beta )}{\alpha -\beta }d\beta \right\| _{L^{2}[-2\delta ,2\delta ]}\\&\quad \lesssim \Vert G(\alpha ,\beta )\Vert _{C^k([-2\delta ,2\delta ]\times \mathbb {T})}\Vert g\Vert _{H^{k-1}(\mathbb {T})} \end{aligned}$$

### Proof

We have

$$\begin{aligned}&\partial _{\alpha }^{k}\left( \int _{-\pi }^{\pi }G(\alpha ,\beta )\frac{g(\alpha )-g(\beta )}{\alpha -\beta }d\beta \right) -\int _{-\pi }^{\pi }G(\alpha ,\beta )\frac{\partial _{\alpha }^{k}g(\alpha )-\partial _{\beta }^{k}g(\beta )}{\alpha -\beta }d\beta \\&\quad =\sum _{1\le j\le k} C_j\int _{-\pi }^{\pi }\partial _{\alpha }^{j}(G(\alpha ,\alpha -\beta ))\frac{\partial _{\alpha }^{k-j}g(\alpha )-\partial _{\beta }^{k-j}g(\alpha -\beta )}{\beta }d\beta . \end{aligned}$$

Then for $j\le k-1$. we could use Lemma 11.2 to get the estimate. Then for $j= k$. we could use Lemma 11.4 to get the estimate. $\square$

### Lemma 11.7

For $g(\alpha )\in H^3(\mathbb {T})$, $h(\alpha )\in H^3(\mathbb {T})$, $G(\alpha ,\beta )\in C^3([-2\delta ,2\delta ]\times \mathbb {T})$, we have

$$\begin{aligned}&\left\| \partial _{\alpha }^{3}\left( \int _{-\pi }^{\pi }G(\alpha ,\beta ) \frac{g(\alpha )-g(\beta )}{\alpha -\beta }\frac{h(\alpha )-h(\beta )}{\alpha -\beta }d\beta \right) \right\| _{L^2[-2\delta ,2\delta ]}\\&\quad \lesssim \Vert G(\alpha ,\beta )\Vert _{C^3([-2\delta ,2\delta ] \times \mathbb {T})}\Vert g\Vert _{H^{3}(\mathbb {T})}\Vert h\Vert _{H^{3}(\mathbb {T})}. \end{aligned}$$

### Proof

We take the derivative and have

$$\begin{aligned}&\partial _{\alpha }^{3}(\int _{-\pi }^{\pi }G(\alpha ,\alpha -\beta )\frac{g(\alpha )-g(\alpha -\beta )}{\beta }\frac{h(\alpha )-h(\alpha -\beta )}{\beta }d\beta )\\&\quad =\sum _{j_1+j_2+j_3=3}\int _{-\pi }^{\pi }\partial _{\alpha }^{j_1}(G(\alpha ,\alpha -\beta ))\frac{\partial _{\alpha }^{j_2}g(\alpha )-\partial _{\alpha }^{j_2}g(\alpha -\beta )}{\beta }\frac{\partial _{\alpha }^{j_3}h(\alpha )-\partial _{\alpha }^{j_3}h(\alpha -\beta )}{\beta }d\beta . \end{aligned}$$

If $$j_2\le 1$$, $$j_3\le 1$$, then

$$\begin{aligned}&\left\| \int _{-\pi }^{\pi }\partial _{\alpha }^{j_1}(G(\alpha ,\alpha -\beta )) \frac{\partial _{\alpha }^{j_2}g(\alpha )-\partial _{\alpha }^{j_2}g(\alpha -\beta )}{\beta } \frac{\partial _{\alpha }^{j_3}h(\alpha )-\partial _{\alpha }^{j_3}h(\alpha -\beta )}{\beta }d\beta \right\| _{L^2[-2\delta ,2\delta ]}\\&\quad \lesssim \Vert \partial _{\alpha }^{j_1}(G(\alpha ,\alpha -\beta ))\Vert _{C^0([-2\delta ,2\delta ]\times \mathbb {T})}\Vert g\Vert _{C^{2}(\mathbb {T})}\Vert h\Vert _{C^{2}(\mathbb {T})}\\&\quad \lesssim \Vert G(\alpha ,\beta )\Vert _{C^3([-2\delta ,2\delta ]\times \mathbb {T})}\Vert g\Vert _{H^{3}(\mathbb {T})}\Vert h\Vert _{H^{3}(\mathbb {T})}. \end{aligned}$$

If $j_2=3$, then $j_1=j_3=0$, by Lemma 11.3, we have

$$\begin{aligned}&\left\| \int _{-\pi }^{\pi }\partial _{\alpha }^{j_1}(G(\alpha ,\alpha -\beta )) \frac{\partial _{\alpha }^{j_2}g(\alpha )-\partial _{\alpha }^{j_2}g(\alpha -\beta )}{\beta } \frac{\partial _{\alpha }^{j_3}h(\alpha )-\partial _{\alpha }^{j_3}h(\alpha -\beta )}{\beta }d\beta \right\| _{L^2[-2\delta ,2\delta ]}\\&\quad \lesssim \Vert (G(\alpha ,\alpha -\beta ))\Vert _{C^1([-2\delta ,2\delta ]\times \mathbb {T})}\Vert g\Vert _{H^{3}(\mathbb {T})}\Vert h\Vert _{C^{2}(\mathbb {T})}\\&\quad \lesssim \Vert G(\alpha ,\beta )\Vert _{C^3([-2\delta ,2\delta ]\times \mathbb {T})}\Vert g\Vert _{H^{3}(\mathbb {T})}\Vert h\Vert _{H^{3}(\mathbb {T})}. \end{aligned}$$

If $j_2=2$, then $j_1=1$ or $j_3=1$, by Lemma 11.4, we have

$$\begin{aligned}&\left\| \int _{-\pi }^{\pi }\partial _{\alpha }^{j_1}(G(\alpha ,\alpha -\beta )) \frac{\partial _{\alpha }^{j_2}g(\alpha )-\partial _{\alpha }^{j_2}g(\alpha -\beta )}{\beta } \frac{\partial _{\alpha }^{j_3}h(\alpha )-\partial _{\alpha }^{j_3}h(\alpha -\beta )}{\beta }d\beta \right\| _{L^2[-2\delta ,2\delta ]}\\&\quad \lesssim \Vert \partial _{\alpha }^{j_1}(G(\alpha ,\alpha -\beta ))\Vert _{C^0([-2\delta ,2\delta ]\times \mathbb {T})}\Vert g\Vert _{H^{3}(\mathbb {T})}\Vert \partial _{\alpha }^{j_3}h\Vert _{C^{1}(\mathbb {T})}\\&\quad \lesssim \Vert G(\alpha ,\beta )\Vert _{C^3([-2\delta ,2\delta ]\times \mathbb {T})}\Vert g\Vert _{H^{3}(\mathbb {T})}\Vert h\Vert _{H^{3}(\mathbb {T})}. \end{aligned}$$

When $j_3=3$ or $j_3=2$, it can treated similarly as $j_2=3$ and $j_2=2$. $\square$
